# The autoinhibited state of MKK4: Phosphorylation, putative dimerization and R134W mutant studied by molecular dynamics simulations

**DOI:** 10.1016/j.csbj.2020.09.017

**Published:** 2020-09-20

**Authors:** Ekaterina Shevchenko, Antti Poso, Tatu Pantsar

**Affiliations:** a Dept of Internal Medicine VIII, University Hospital Tübingen, Otfried-Müller-Strasse 14, 72076 Tübingen, Germany; b School of Pharmacy, University of Eastern Finland, Yliopistonranta 1C, 70210 Kuopio, Finland; c Department of Pharmaceutical and Medicinal Chemistry, Institute of Pharmaceutical Sciences, Eberhard Karls Universität Tübingen, Auf der Morgenstelle 8, 72076 Tübingen, Germany

**Keywords:** Protein kinases, MAP kinase kinase 4, Molecular dynamics simulation, Protein conformation, Protein dimerization

## Abstract

Protein kinases are crucial components of the cell-signalling machinery that orchestrate and convey messages to their downstream targets. Most often, kinases are activated upon a phosphorylation to their activation loop, which will shift the kinase into the active conformation. The Dual specificity mitogen-activated protein kinase kinase 4 (MKK4) exists in a unique conformation in its inactive unphosphorylated state, where its activation segment appears in a stable α-helical conformation. However, the precise role of this unique conformational state of MKK4 is unknown. Here, by all-atom molecular dynamics simulations (MD simulations), we show that this inactive state is unstable as monomer even when unphosphorylated and that the phosphorylation of the activation segment further destabilizes the autoinhibited α-helix. The specific phosphorylation pattern of the activation segment has also a unique influence on MKK4 dynamics. Furthermore, we observed that this specific inactive state is stable as a dimer, which becomes destabilized upon phosphorylation. Finally, we noticed that the most frequent MKK4 mutation observed in cancer, R134W, which role has not been disclosed to date, contributes to the dimer stability. Based on these data we postulate that MKK4 occurs as a dimer in its inactive autoinhibited state, providing an additional layer for its activity regulation.

## Graphical abstract

**Figure:**
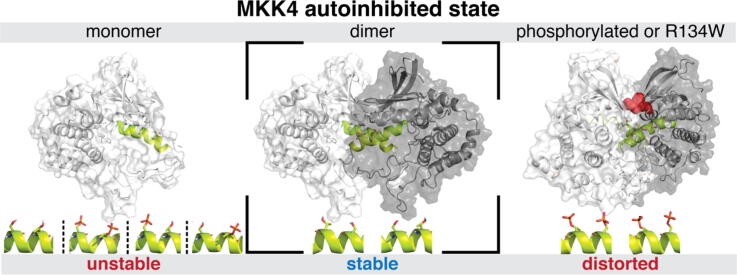

## 1 Introduction

One of the key processes in the regulation of complex cellular signalling networks is protein phosphorylation [1]. This phosphorylation is conducted by protein kinases, which transfer a phosphate from an ATP molecule to a target protein, modulating the activity of their downstream target. To maintain the cellular homeostasis, kinase activity is under a strict regulation in the cells. This highly regulated kinase activity is severely distorted in a wide variety of diseases, such as in cancer, resulting in deregulated cellular signalling and disease progression [2], [3]. Already several kinase inhibitors are available in the clinical use with the main indication in cancer and oncology, while emerging therapeutic areas include autoimmune and inflammatory diseases [4]. As the therapeutic potential of the protein kinases is enormous, there is a growing need to understand the function and behaviour of these dynamic proteins and their subtle discrepancies in more detail.

Protein kinases not only modulate their target proteins’ activity by phosphorylation, but also kinases’ activity itself is most often regulated via phosphorylation [5]. Most of the kinases are phosphorylated in their activation loop that transform the kinase from its inactive state to its active state, where the kinase is able to bind and phosphorylate its substrate(s). Interestingly, some kinases have a secondary phosphorylation site in close proximity to the primary site in their activation loop. For instance, two phosphorylation sites are found in ERK2 (THR183/TYR185) with one-residue in between [6], whereas CHK2 (THR383/THR387) exhibits three-residues in between [7], [8], while SLK (THR183/SER189) and LOK (THR185/SER191) kinases show a five-residue distance in their activation loop phosphorylation sites [8], [9]. Although dual phosphorylation is required, for instance, for full activation of ERK2 [6], the exact result from different phosphorylation patterns of two nearby phosphorylation sites is generally not well understood with most of the kinases. Furthermore, protein kinases may exploit dimerization and/or multimerization as a way to control their kinase activity [10]. For example, kinase dimerization is related to active state with CHK2 [11], DAPK3 [8], RAF kinases and RIPK1 [12], [13], [14], [15], whereas inactive dimers exist, *e.g.,* with ligand-free EGFR (inactive symmetric kinase domain dimer) and PDZ-binding kinase (PBK/TOPK) [16], [17], [18], [19].

One of the kinases, which has two phosphorylation sites in the close proximity of each other in its activation segment, is the dual specificity mitogen-activated protein kinase kinase 4 (MKK4). MKK4 is encoded by the *MAP2K4* gene, consist of 399 residues and has one isoform with 97.3% identity (410 residues, with an 11 residue insert in the N-terminal part). MKK4 has a serine (SER257) and a threonine (THR261) residues in its activation segment that can be phosphorylated [20]. The phosphorylation of the SER257 is essential for MKK4′s activation, but the THR261 phosphorylation is required for its full activation [21]. The dual specificity name originates from the fact that MKK4 is able to phosphorylate and activate both, c-Jun NH2-terminal kinases (JNKs) and p38 MAP kinases, displaying preference in phosphorylating tyrosine in JNKs activation loop and threonine in the latter [22]. In turn, activated JNKs and p38 MAP kinases are involved in such biological processes like proliferation, apoptosis and cell differentiation [23]. Not much is known about possible dimerization of MKK4.

MKK4 has been suggested to play a crucial role in certain physiological functions and disease development; notably, it has a decisive function in liver regeneration [24]. In tumour development, its role is somewhat controversial, or at least appears to be tissue dependent. Generally, however, MKK4 is considered as a tumour suppressor [25]. Inactivation of MKK4 can exert tumour suppressor activity at both early and late stages of lung tumorigenesis [26]. Also, decreased expression of MKK4 is related to ovarian cancer metastasis and its downregulated phosphorylation levels are associated with a poor prognosis in colorectal cancer patients [27], [28]. Conversely, increased activity of MKK4 was shown to promote prostate cancer [29]. In tumours across the primary tissue types, MKK4 is underexpressed in the ovary (84.21%), in the large intestine (23.28%) and in the pancreas (21.23%) [30], [31]. Loss of function mutations in the MKK4 gene were reported in approximately 5% of tumours from a variety of tissues [32]. Moreover, it was recently noted as a significantly mutated gene in the colorectal cancer [33]. Based on COSMIC database, 2% of all tumours harbour MKK4 mutation, highlighting two hot-spot mutations: R134W and S184L (COSMIC v.91) [31]. The S184L is located in the ATP-binding site, most likely compromising nucleotide binding, and is an inactivating mutation [34]. The most frequent MKK4 mutation R134W, where an arginine residue is replaced with a tryptophan, is located in the loop between β3-sheet and αC-helix. However, no functional data of the R134W mutation exist to date and its effect on MKK4′s function is unclear.

Currently three MKK4 structures are available in the RCSB Protein Data Bank (PDB IDs: 3alo [35], 3aln [35], 3vut [36]) (SI Fig. S1). All of the structures represent the inactive unphosphorylated form of the protein, where 3vut is the apo-structure and 3aln and 3alo are co-crystallized with the non-hydrolysable ATP analogue, AMP-PNP. In addition, 3alo (resolution 2.6 Å, R_free_ 0.271) is crystallized with a short p38 peptide that is bound on top of the N-terminal lobe. This structure is particularly interesting, as a part of its activation segment (ILE250–ARG264) appears in an ordered autoinhibited conformation, forming a long α-helix that protrudes from the kinase (SI Fig. S1). The other structures, 3aln (resolution 2.3 Å, R_free_ 0.378) and 3vut (resolution 3.5 Å, R_free_ 0.407), are lower quality with two disordered regions: ASP263–GLY283 and GLN316–VAL320. Furthermore, 3vut is missing its C-terminal part after ALA374.

Not much is known of MKK4′s conformational dynamics. Recently, MKK4 was studied by small angle X-ray scattering (SAXS) with Ensemble Optimization Method (EOM) [37], revealing an ensemble of conformations in solution for all three structures. Only one study has been reported to date, where MD simulations were applied to investigate MKK4 [38]. In the study, a homology model based on 3aln structure was used and a single 400 ns simulation for wild-type MKK4 and G265D mutant was conducted. Overall, there is a lack of knowledge in the MKK4 dynamics, especially related to the specific autoinhibited state.

Here, we conducted microsecond timescale (a total of 40 μs) all-atom MD simulations to study the dynamics of autoinhibited MKK4. We studied the role and influence of all phosphorylation patterns in the activation segment. Finally, we investigated putative dimerization of MKK4 and the effect of phosphorylation to the stability of the homodimer, including the most common MKK4 mutant R134W to these simulations. Our results highlight the instability of the autoinhibited state as a monomer and suggest that it may exist as a stable dimer only when unphosphorylated.

## 2 Methods

### 2.1 MD simulations of monomer MKK4

For the simulations we used the autoinhibited MKK4 structure 3alo [35]. System preparation was done with Maestro 2017-2 (Schrödinger, LLC, New York, NY) with OPLS3 force field [39]. The disordered residues of the activation segment in the 3alo structure (residues SER277–GLY283) were added with Maestro’s *cross link proteins* tool. First, the PRO277 residue was deleted, the rotamer of TYR284 was changed (to prevent the clash) and the sequence PRO277–GLY283 (PSASRQG) was added to link the chain. The terminal PRO389 was mutated to ALA as a terminal PRO residue distorts the dynamics (the C-terminal part ALA390–ASP399 that is missing from the structure was omitted). The systems were prepared with Protein Preparation Wizard with default settings (Cap-termini) [40]. We left out the disordered N-terminal part of MKK4 (residues 1–94), which is suggested to play a role in substrate recognition [41] and therefore was not considered critical for our simulations. For the phosphorylated systems, SER257 and THR261 were changed to the corresponding phosphoresidues pSER257 and pTHR261 [42], [43].

Desmond MD engine was used for the simulations [44]. Systems were solvated in a cubic box (minimum distance of 13 Å to the edges from the protein), and the total net charge was neutralized using Na^+^-ions. The water was described with TIP3P model [45]. The final systems comprised ~59 k atoms. All simulations were run in NpT ensemble (T = 310 K, Nosé-Hoover method; p = 1.01325 bar, Martyna-Tobias-Klein method) with default Desmond settings. RESPA integrator with 2 fs, 2 fs and 6 fs timesteps were used for bonded, near and far, respectively. The default value of 9 Å was used for Coulombic cut-off. All systems were relaxed using the default Desmond protocol prior to the production simulations. To obtain better sampling and to remove the potential initial bias in the systems, the ARG134 rotamer was changed to different ones for each replica (according to the rotamer library) as it was pointing towards the phosphoresidues in its initial configuration (in the unphosphorylated crystal structure there is no clear electron density for the side-chain of ARG134). Five replicas of production simulations were carried out for each system for 1000 ns (5 × 4 × 1000 ns = 20 μs). For each replica, a random seed was used. All replica simulations were run using OPLS3 force field, except three individual replicas were run with updated OPLS3e force field (using Maestro 2018-2) [46].

### 2.2 MD simulations of dimer MKK4

The dimer assembly (lowest energy assembly) was obtained with PDBePISA server (v.1.52) [47]. The dimer complexes were prepared as monomers, except the force field OPLS3e [46] was used (with Schrödinger Maestro 2018-2 and 2019-3). For the dimer simulations the cubic box was set to 15 Å from the protein. Final systems comprised ~120 k atoms. Five replicas of production simulations were carried out with same settings as mentioned above. Each system was simulated for 1000 ns (5 × 4 × 1000 ns = 20 μs). For each replica a random seed was used.

### 2.3 RMSD and RMSF

Root-mean-square fluctuations (RMSFs) of protein backbone and Root-mean-square deviations (RMSDs) of Cα-atoms were calculated using Maestro Simulation interaction analysis tool (Schrödinger, LLC, New York, NY). RMSDs of residue intervals used for angle calculations were conducted with MDAnalysis.analysis.rms module [48] of MDAnalysis library [49], [50] for Python 3.7.

### 2.4 Principal component analysis (PCA)

The PCA was conducted with GROMACS (version 2019) covariance analysis tools (gmx covar, gmx anaeig) [51]. The PCA was conducted for all backbone atoms, excluding the residues SER257 and THR261 which differed among the systems (as a single PCA was conducted for all systems). For the further analysis we included the PCs that displayed >9% individual contribution: PC1 20.0%; PC2 11.8%; PC3 9.4% and PC4 9.1% (all combined 50.3%). The individual PC movements were illustrated with PyMOL-script Modevectors [52].

### 2.5 Secondary structure analysis

Secondary structure analysis was conducted with Maestro Simulation interaction analysis tool (Schrödinger, LLC, New York, NY). The percentage of secondary structure elements (%SSE) throughout the simulations was calculated with Python 3.7.

### 2.6 Angle calculations

Angle calculations between subunits of MKK4 were conducted using open-source MDAnalysis library for Python 3.7 [49], [50]. For both monomer and dimer systems, first 250 ns were excluded from the analysis based on the system stabilization (SI Fig. S2–4). Sides of the angle are formed by: ILE250–ALA264 (ASH), GLU139–ARG154 (αC-Helix), the vertex is represented by GLU179–SER182 (HR). Calculated centre of geometry (cog) was used as an apex point for the angle calculations. The data from all frames for each system replica was combined and used for calculation of average (mean) and standard deviation for both mono- and dimer system of MKK4. In dimer system calculations were performed individually for both subunits A and B. Reference angles were calculated from frame 0 for dimer systems that are 14.9° and 17.9° for subunit A and B, respectively. For monomer systems 14.9° (subunit A) was chosen as reference value, that corresponds to the autoinhibited crystal conformation.

In order to confirm that the selection of particular ASH residues is not critical for angle value, we performed validation by testing different intervals. Residues ILE250–ALA264 (ASH), that are forming one side of the angle, were switched to ILE250–ALA259 and VAL255–ALA264 (SI Fig. S5). This way we shifted the selected residue interval of ASH by five residues back and forth. When compared to the switched ones, ILE250–ALA259 interval showed deviation of - ~1–2° from reference and VAL255–ALA264 of + ~1–2°. As a result, the selection of the residues itself do not play a critical role on the angle value as the two rays lie in a plane, but this plane does not have to be Euclidian one.

### 2.7 Distance calculations

The distances between subunits of MKK4 were calculated using GROMACS (version 2019) gmx distance tool [51]. Points for calculation were defined by cog of selected residue intervals. Following residues of each subunit were used for cog calculations: LEU102–GLN126 (N-lobe section), ILE250–ALA264 (ASH), VAL286–THR302 (αF-helix). The choice of this particular residues was based on that each of these intervals represent a specific region within MKK4 interface. Thus, N-terminal lobe, includes allosteric region, that predominantly bound the p38a peptide [35]; activation segment includes two phosphorylation sites of MKK4 (SER257, THR261), and αF-helix represents stable helix in lower part of C-lobe. Consequently, distance of the cog for chosen intervals represent three points within the dimer interface: upper, middle and lower part, where the middle part distance is perpendicular to others.

Distance calculations within the N-lobe MKK4 were done as above, using the following residue intervals of each subunit: VAL116–VAL120; VAL120–ILE127; PHE164–LEU168; ALA111–GLY114.

### 2.8 Interaction analysis

The interaction networks analysis related to Fig. 4 was conducted with Maestro (Schrödinger, LLC, New York, NY) scripts, analyse_trajectory_ppi.py for salt-bridges and trajectory_asl_monitor.py for hydrophobic interactions. Default salt-bridge interaction cut-off of 4.0 Å was used. For hydrophobic interaction definition, we used a sidechain atom distance below 2.5 Å. Residue 134 interaction analysis related to Fig. 7 was conducted with simulation interaction analysis tool of Maestro.

### 2.9 Data visualization

Results were plotted with Seaborn library for Python 3.7 [53]. Protein structures were visualized with PyMOL (The PyMOL Molecular Graphics System, Version 2.0 Schrödinger, LLC.) Graphical representations of figures were arranged using Adobe Illustrator©. Supplementary movies were generated with PyMOL.

## 3 Results

### 3.1 Conformational dynamics of monomeric autoinhibited MKK4 with different phosphorylation states

#### 3.1.1 MKK4's activation segment and C-lobe loop are highly dynamic

**Fig. 1:**
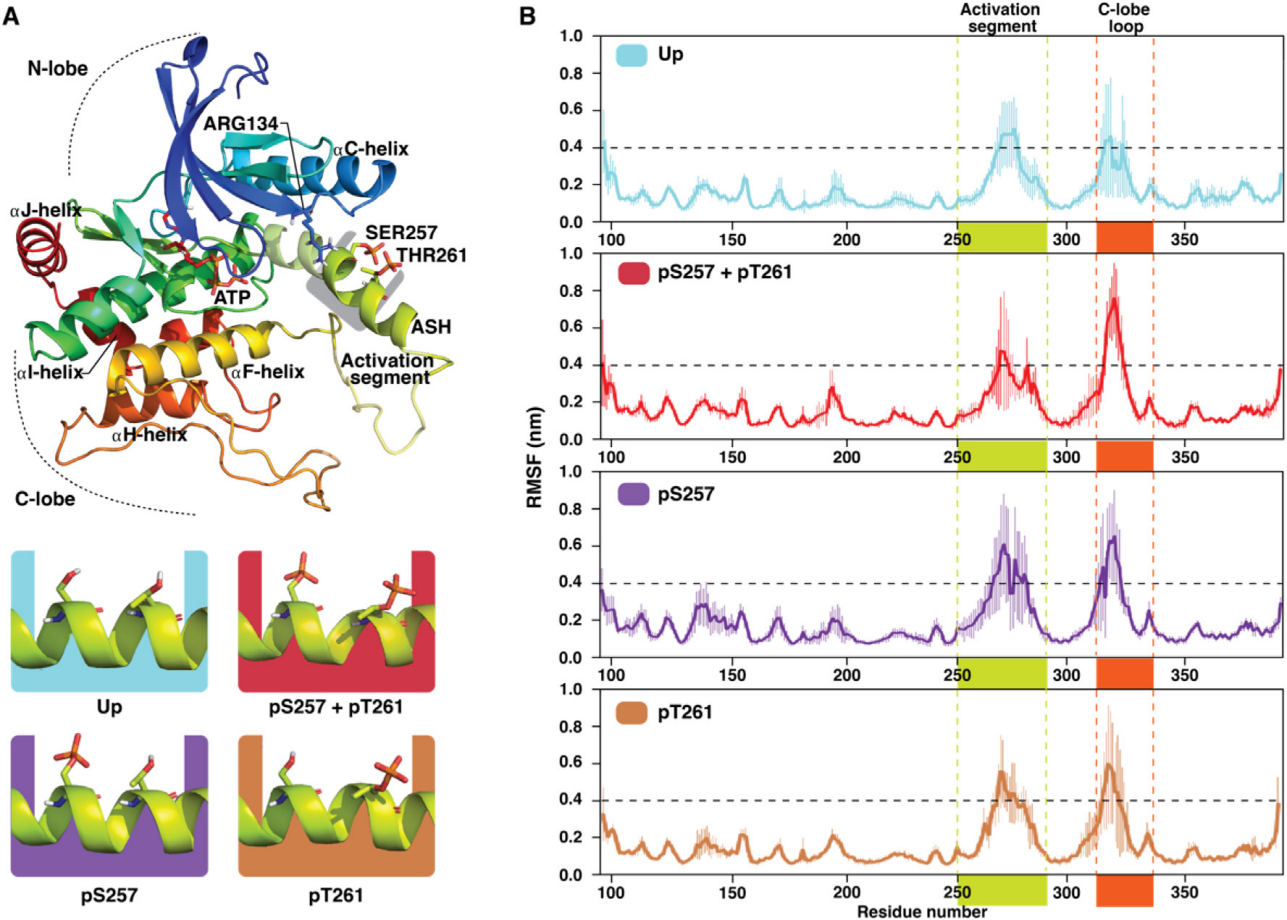
Structure and dynamics of MKK4 with different phosphorylation states in its autoinhibited state. (A) Conformation of MKK4 in its autoinhibited state (PDB ID: 3alo). In its autoinhibited state, part of the activation (loop) segment is forming a helical structure; activation segment helix (ASH). Different phosphorylation patterns in its activation segment are colour coded throughout this article as: Up, teal; pS257 + pT261, red; pS257, violet; pT261, brown. (B) Root-mean-square fluctuation (RMSF) of protein backbone. Average of five replicas is shown with standard deviation (thin vertical lines). Highlighted regions, represented in same colours as in A, indicate activation segment (ILE250–SER292), green; C-lobe loop (PRO308–PHE340), dark orange. RMSF value of 0.4 nm is indicated with the horizontal dashed black line. (For interpretation of the references to colour in this figure legend, the reader is referred to the web version of this article.)

First, we investigated dynamics of autoinhibited MKK4 with all possible phosphorylation patterns in its activation segment (a total of 20 μs MD simulations). Four different phosphorylation states of MKK4 in this region are possible: unphosphorylated (Up), monophosphorylated at SER257 (pS257) or at THR261 (pT261) and double phosphorylated (pS257 + pT261) (Fig. 1A). Overall, these systems exhibit similar root-mean-square fluctuation (RMSF) values (Fig. 1B). All systems display the highest RMSF-values at two specific loop-regions, particularly among the residues CYS266–ARG281 of the activation segment and residues ASP315–LYS322 in a loop of the C-lobe. Generally, kinases have αG-helix located on this C-lobe loop location [5], but it appears as a loop in available MKK4 structures. We next compared how the observed dynamics based on RMSF is in agreement with observed B-factors and disorder of the MKK4 crystal structure. Indeed, disorder (residues 278–283) or high B-factor values are observed in these regions (SI Fig. S6). Although generally a similar trend in RMSF values is observed among all systems, slightly different RMSF patterns are evident (see details in SI Table S1). For instance, higher RMSF (>0.1 nm compared to Up) of SER251–ALA259 is observed only in monophosphorylated pS257. This indicates an individual change in dynamics related to particular phosphorylation state of MKK4.

#### 3.1.2 Phosphorylation defines MKK4 dynamics

**Fig. 2:**
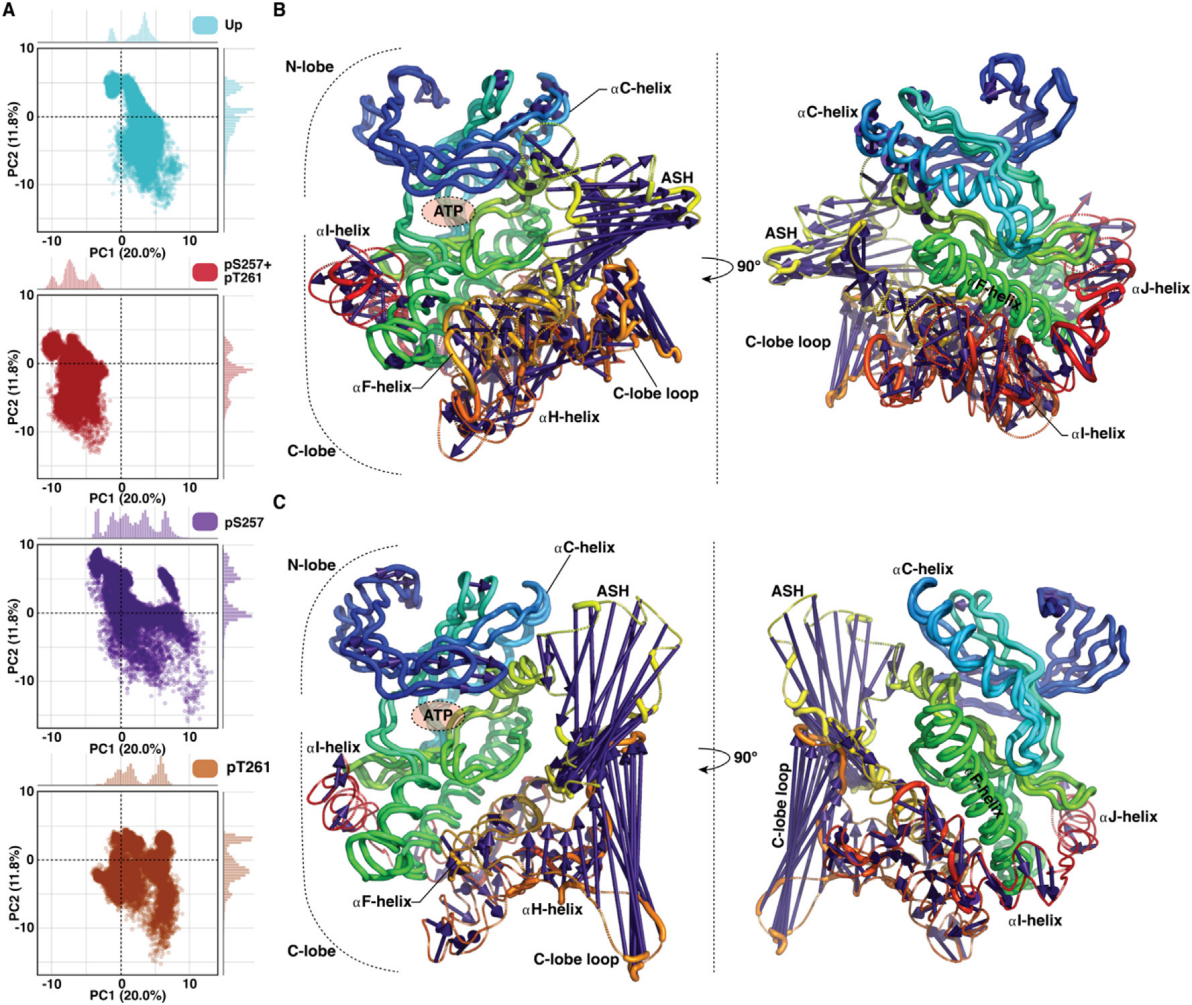
MKK4 shows individual conformational dynamics with different phosphorylation states. (A) Principal component analysis (PCA) score plot of PC1 and PC2. A single PCA was conducted for all systems, ensuring the comparability of the score-plots. (B) The extreme movements of PC1. (C) The extreme movements of PC2. In B and C, protein is illustrated with rainbow colour (as in Fig. 1A) and the purple arrows indicate the extreme movements related to each principal component. (For interpretation of the references to colour in this figure legend, the reader is referred to the web version of this article.)

To gain further insights into the protein dynamics and possible differences among the systems, we conducted principal component analysis (PCA). According to the PCA phosphorylation affects to the MKK4 dynamics, as each system displays individual profile in their PC (principal component) score plots (Fig. 2A). In PC1 the largest movements appear in activation segment and C-lobe loop, which also displayed the highest RMSF values (Fig. 2B, SI Movie M1). Moreover, considerable movement is observed in other regions of the protein: αC-helix and in C-lobe helices αF, αH, αI and αJ. Clearly lower values of PC1 are observed with the pS257 + pT261 in comparison to other systems. This indicates that the protruding movement of the activation segment (elongation of the ASH towards solvent *i.e.* original crystal structure conformation) is clearly disfavoured with the double phosphorylated system. In PC2, movements of activation segment and C-lobe loop are dominating (Fig. 2C, SI Movie M2). Both are folding towards the centre of the kinase. With this component the monophosphorylated pS257 system shows the highest values (Fig. 2A). With PC3 and PC4 (SI Fig. S7, SI Movies M3–4), pT261 is showing a unique subpopulation with high PC3 and low PC4 values. Overall, the areas displaying the highest contribution to the PCs occur in the regions that are responsible of the activation, substrate binding and regulatory actions in the kinase [54].

#### 3.1.3 Activation segment helix movement is defined by its phosphorylation

In addition to the differences among the systems observed by RMSF and PCA, a system-specific movement is evident even by visual examination of the simulation trajectories. The activation segment appears to display a system specific shift that clearly deviates from the crystal conformation. This is also demonstrated by the resulting end conformations of the simulations (SI Fig. S8, SI Movie M5). Moreover, the intactness of the α-helical secondary structure in the activation segment deviates among the systems (SI Fig. S9).

**Fig. 3:**
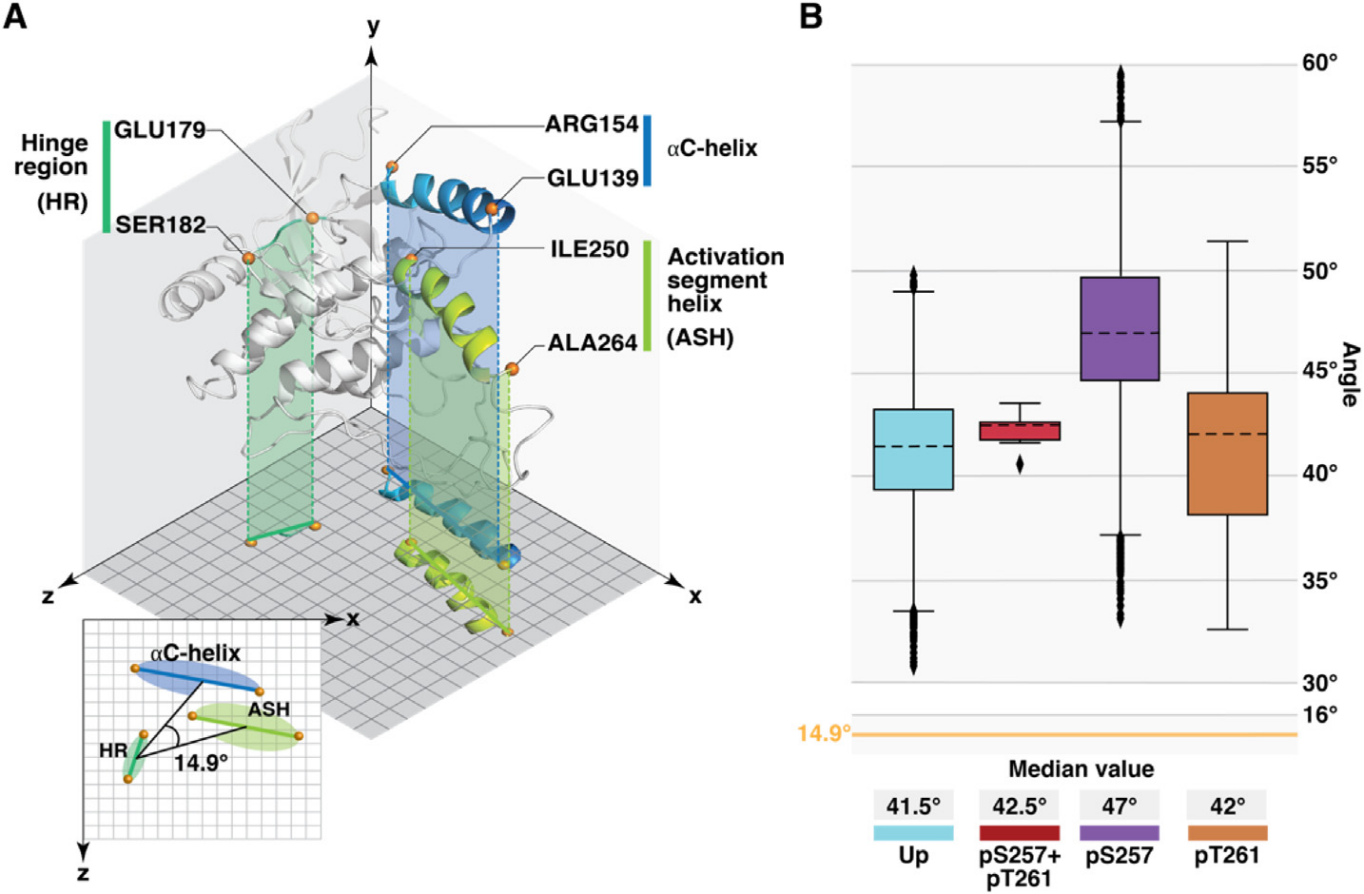
Activation segment helix movement depends on its phosphorylation status. (A) Plane projection of residue intervals used for angle calculations. The change in the angle value describes a relative movement of these elements in respect to others. Apex points of the angle are represented by centre of geometry (cog) for each of the selected residue intervals throughout simulation time. *Cog* is visualised on the plane projection with semi-transparent ovals of the same colours as displayed residue intervals. Reference angle value (14.9°) corresponds to the autoinhibited crystal conformation. (B) Boxplot representation of observed angle values in MKK4 systems with different phosphorylation patterns (excluding the data of the first 250 ns of each simulation). The dashed black line in the box represents the median. Box displays the quartiles of the dataset (25–75%) and whiskers the rest of the data within 1.5 times of the interquartile range (IQR). Outliers are indicated with black diamonds. Reference angle value (14.9°) of the autoinhibited state is indicated with yellow line. (For interpretation of the references to colour in this figure legend, the reader is referred to the web version of this article.)

To validate these visual observations, we further evaluated this movement by angle calculations (Fig. 3; SI Fig. S4). This allowed us to describe the activation segment movement in easily interpretable geometrical values. For these angle calculations, we applied centre of geometry (cog) of selected protein segments: activation segment helix (ASH; ILE250–ALA264), αC-helix (GLU139–ARG154) and hinge region (HR; GLU179–SER182). As hinge region and αC-Helix are relatively stable elements of the kinase, this angle-change provides information about the movement of ASH in respect to the protein and filters out their synchronized fluctuations. For instance, in monophosphorylated pS257 system ASH is constantly fluctuating towards the C-lobe, away from the αC-helix. This movement is directly related to the angle: the more down along the Y-axis ASH moves, the wider the angle (Fig. 3A). Remarkably, none of the systems stay in the autoinhibited crystal conformation (reference angle 14.9°) (Fig. 3B). The highest variation of this angle exists with pS257 (~37–56°), which reflects to the visually observed high fluctuation of the ASH with this system. Interestingly, the double phosphorylation appears to fix MKK4 in a more specific configuration, as clearly less variation in the angle is observed with pS257 + pT261 system. To note, the beginning of ASH is relatively stable in all systems (Fig. 1B); therefore, even small angle variations indicate a considerable movement in the end part of ASH. We anticipate that the higher overall angle values of pS257 system are indeed related to the increased movement of the SER251–ALA259 residues, for which it shows higher RMSF values compared to other systems. These observations highlight clear influence of phosphorylation states on MKK4 dynamics, especially on the ASH region.

**Fig. 4:**
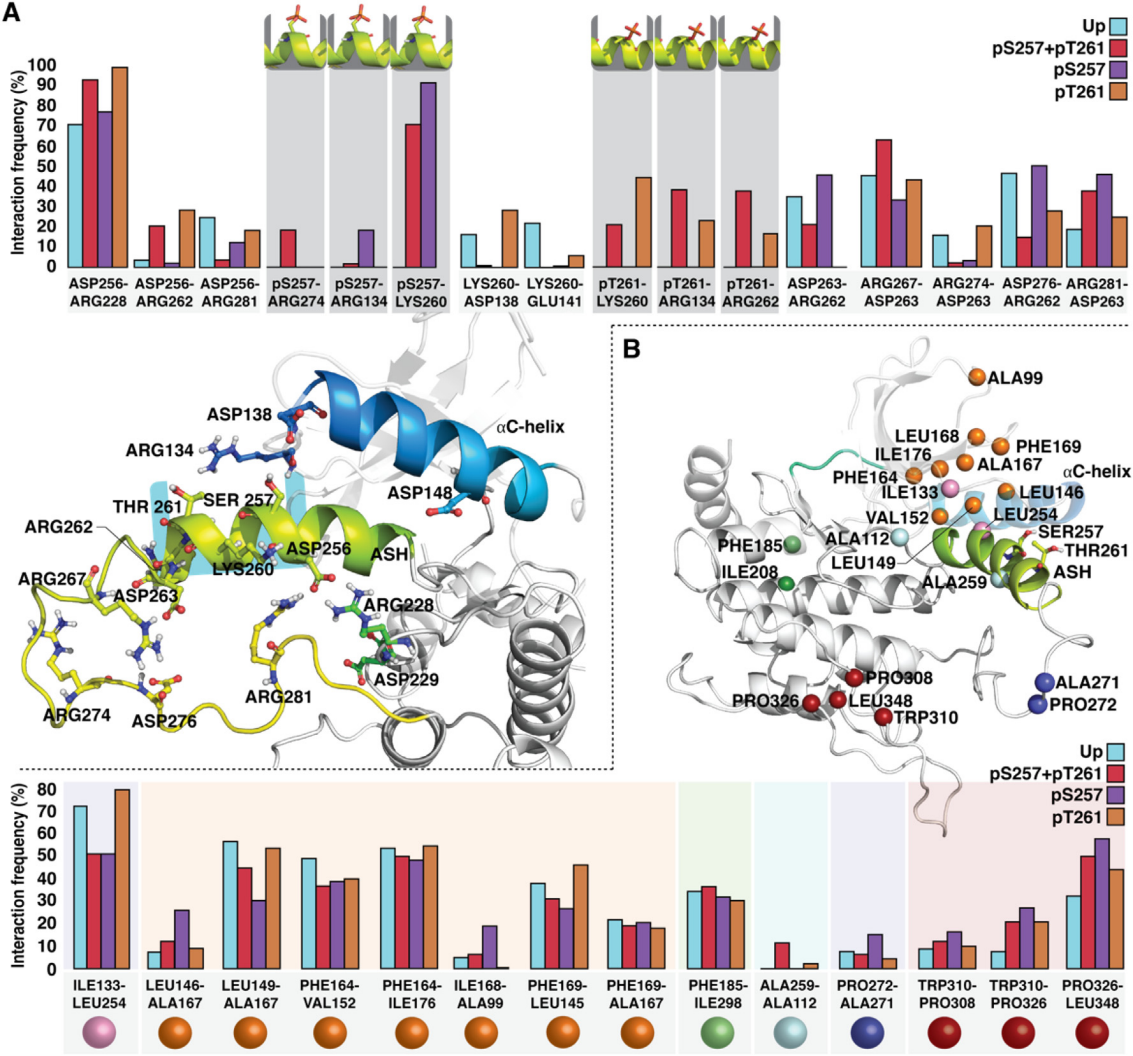
Differences in protein interaction networks among different phosphorylation states. (A) Salt-bridges of the activation segment residues ILE250–ALA264 with their interaction frequencies (%). Only the salt-bridges with > 20% differences in their interaction frequencies among systems are shown. (B) Selected hydrophobic interactions of MKK4 and their interaction frequencies among different systems. The locations of the Cα-atoms of the hydrophobic residues are shown in spheres, which are coloured according to different hydrophobic clusters that exhibit linked interactions.

#### 3.1.4 MKK4 interaction networks are phosphorylation state specific

As phosphorylation status clearly affects MKK4 dynamics, we next conducted interaction network analysis to determine the potential changes in the protein’s interaction patterns. First, we analysed the salt-bridges and their frequencies in the close proximity of the ASH of each individual system (Fig. 4A). Especially in the monophosphorylated system pS257, LYS260 is fixed to the phosphorylated residue (90.4%). In the pS257 + pT261 system, LYS260 seems to be balancing between both of the phosphoresidues; still the major interaction occurring with pS257 (69.3% vs. 21.6%). The monophosphorylated pT261 displays the LYS260–pTHR261 interaction with 43.9% frequency. Interestingly, with pT261 system LYS260 displays 29.7% interaction to ASP138 that is located in the small loop connected to the αC-helix. This interaction is also present with Up (14.8%), but almost missing in pS257 (0.05%) and pS257 + pT261 (0.1%). Contact between LYS260 and ASP138 may explain the lower angle values observed in Up and pT261 systems (Fig. 3B), as it would fix and thus prevent the ASH bending, away from the αC-helix. ARG134, which is located in the same loop with ASP138, interacts with the pTHR261 (21.4% and 38.8% in pT261 and pS257 + pT261 systems, respectively). Interaction between ARG134 and pSER257 is almost non-existing with double phosphorylated system (0.1%), whereas with monophosphorylated system (pS257) it occurs with 15.7%. ARG274 displays interaction with pSER257 only in the double phosphorylated system (18.9%). In addition, ARG228 interacts with the pSER257 in monophosphorylated pS257 system (11.9%), but not in double phosphorylated system (0.4%). Salt-bridge interaction frequencies are also altered among the systems with other residues than phosphoresidues in the ASH.

Systems also display differences in their hydrophobic interactions (Fig. 4B). A specific type of extensive bending of ASH towards G-loop occurs in pS257 + pT261 system, which can be traced to the formation of hydrophobic interaction between ALA259–ALA112 (9.2%). The more stable conformations of the beginning of ASH appearing with Up and pT261 are manifested by the elevated interaction frequencies of ILE133–LEU254. Furthermore, pS257 system displays a clear shift in its interaction preferences in its αC-helix associated hydrophobic residues (orange spheres) in comparison to other systems.

### 3.2 Putative MKK4 homodimer and R134W mutation

#### 3.2.1 MKK4 homodimer

**Fig. 5:**
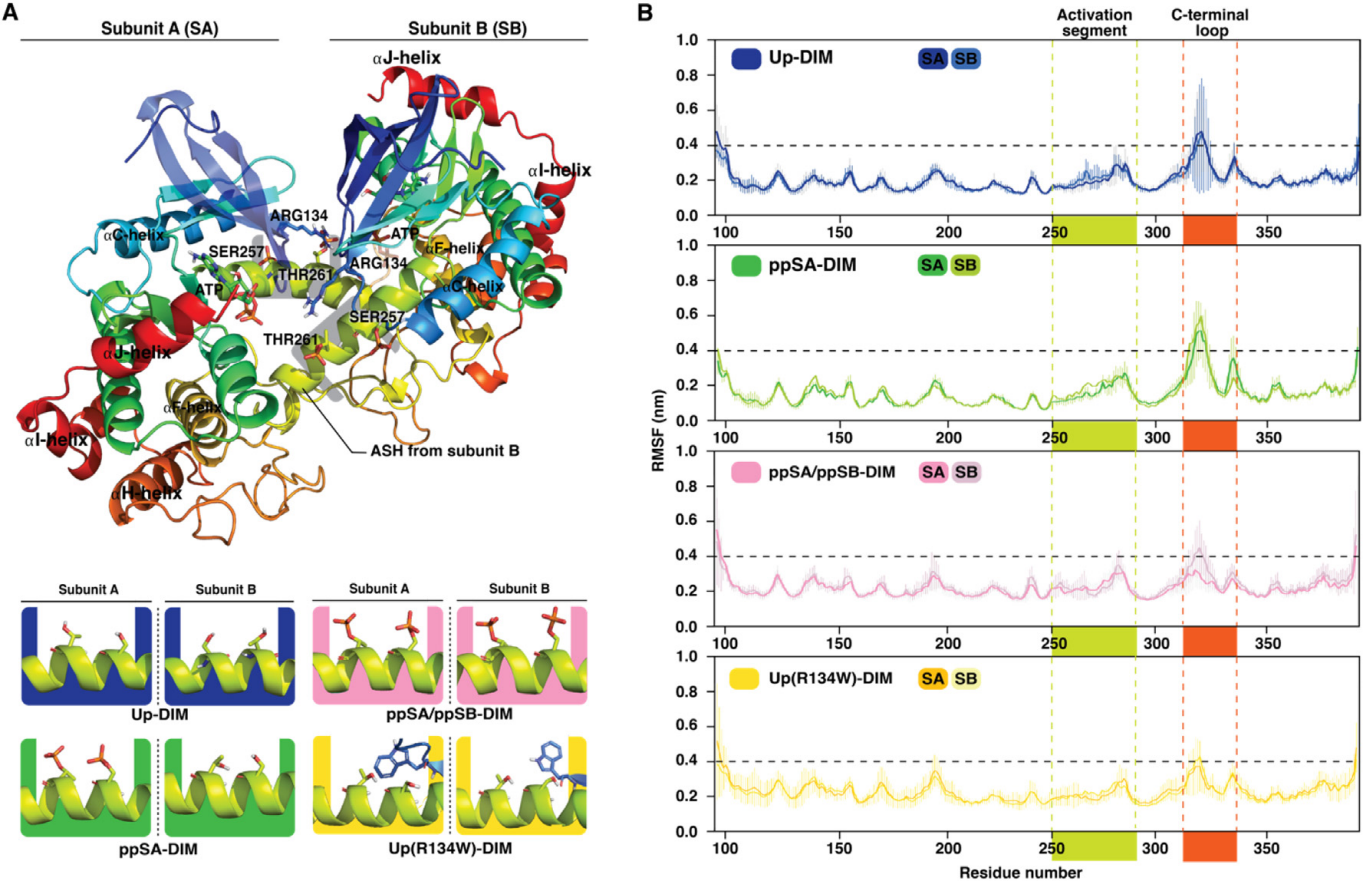
Putative MKK4 homodimer and its dynamics. (A) Assembly of autoinhibited MKK4 homodimer. Phosphorylation patterns in simulated MKK4 dimer systems. Systems are coloured as: Up-DIM, blue; ppSA-DIM, green; ppSA/ppSB-DIM, pink; Up(R134W)-DIM, yellow. (B) Root-mean-square fluctuation (RMSF) of protein backbone. Average of five replicas is shown with standard deviation (thin vertical lines). Highlighted regions indicate activation segment (ILE250–SER292), green; C-lobe loop (PRO308– PHE340), dark orange. RMSF value of 0.4 nm is indicated with the horizontal dashed black line. (For interpretation of the references to colour in this figure legend, the reader is referred to the web version of this article.)

As the autoinhibited MKK4 structure exhibits a long protruding activation segment, which appeared unstable in the monomer simulations, we evaluated the crystal assemblies of 3alo utilizing the PDBePISA server [47]. Indeed, the lowest energy assembly of the structure is identified as a dimer (ΔG^int^ = −53.5 kcal/mol; ΔG^diss^ = 10.3 kcal/mol) and not as a monomer (ΔG^int^ = –23.0 kcal/mol; ΔG^diss^ = 5.2 kcal/mol). In this homodimer assembly the apical activation segment region is stabilized and buried within the dimer interface (Fig. 5A). Therefore, we considered the possibility of MKK4 existing as a dimer in its autoinhibited state and decided to investigate this dimer and its stability by MD simulations. We conducted simulations of dimer MKK4 (a total of 20 μs) with different phosphorylation patterns: unphosphorylated (Up-DIM), double phosphorylated at SER257 and THR261 in one subunit (ppSA-DIM), double phosphorylated in both subunits (ppSA/ppSB-DIM) (Fig. 5A). In addition, as we noticed that the most frequent mutation of MKK4, R134W, is located in the dimer interface, we included unphosphorylated system accompanied with this mutation in both of the subunits (Up(R134W)-DIM).

First, we investigated the overall dynamics of the protein by RMSF. Compared to monomer systems, homodimer MKK4 displays significantly lower RMSF-values in its activation segment (Fig. 5B). This is perhaps not surprising, as the activation segment movements are hindered in the dimer by the other subunit. The C-lobe loop displays more comparable RMSF values with the monomer systems, although those are also generally lower.

#### 3.2.2 MKK4 homodimer is stable only when unphosphorylated

**Fig. 6:**
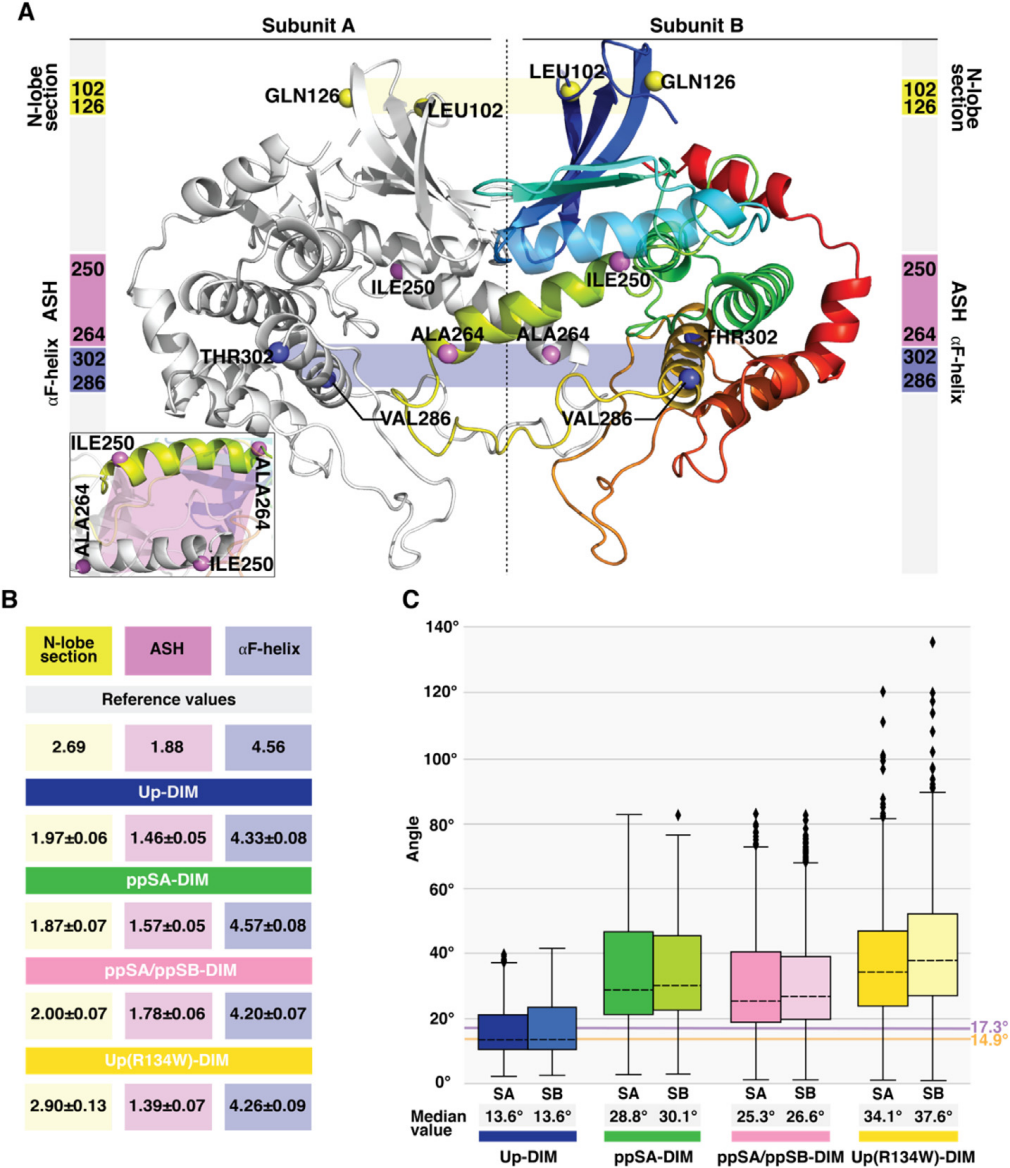
Conformational stability of MKK4 homodimer. (A) Selected residue intervals and their locations for distance calculation between the subunits: LEU102–GLN126 (N-lobe section), ILE250–ALA264 (ASH) and VAL286–THR302 (αF-helix). (B) Average distance (nm) with standard deviation of the selected residue intervals between subunit A and B in MKK4 dimer systems. First 250 ns of the simulations were excluded from the analysis. See more details in SI Table S2. (C) Boxplot representation of αC-helix–HR–ASH angle values in dimer MKK4. Reference values of the corresponding angles are illustrated with orange line for subunit A (14.9°) and violet line for subunit B (17.34°). The black dashed horizontal line in the box represents the median. Box displays the quartiles of the dataset (25–75%) and whiskers the rest of the data within 1.5 times of the IQR. Outliers are indicated with black diamonds. (For interpretation of the references to colour in this figure legend, the reader is referred to the web version of this article.)

Next, we evaluated the stability of the dimer complexes via a distance analysis. Three regions of MKK4 were selected for distance calculation with the following residue intervals: LEU102–GLN126 (N-lobe section), ILE250–ALA264 (ASH), VAL286–THR302 (αF-helix) (Fig. 6A). To note, distance between ASH is perpendicular compared to the other two selected intervals. Distances between αF-helices, which are buried deeply in the dimer interface in the C-lobe, remain close to the reference value of the crystal structure (Fig. 6B; SI Table S2). The ASH distance values indicate even tighter packing in the middle compared to the crystal. Here the shortest distance among wild-type dimer systems is displayed by Up-DIM, where with the unphosphorylated mutant it is even shorter. Overall, N-lobe from different subunits tends to come closer to each other with increase of simulation time among all wild-type systems. Remarkably, with R134W this is not the case; even increased distance compared to the crystal structure is observed. In the N-lobe the unphosphorylated mutant displays a striking difference compared to other systems (0.93 nm compared to Up-DIM), having the longest distance (2.90 nm) between N-lobes.

**Fig. 7:**
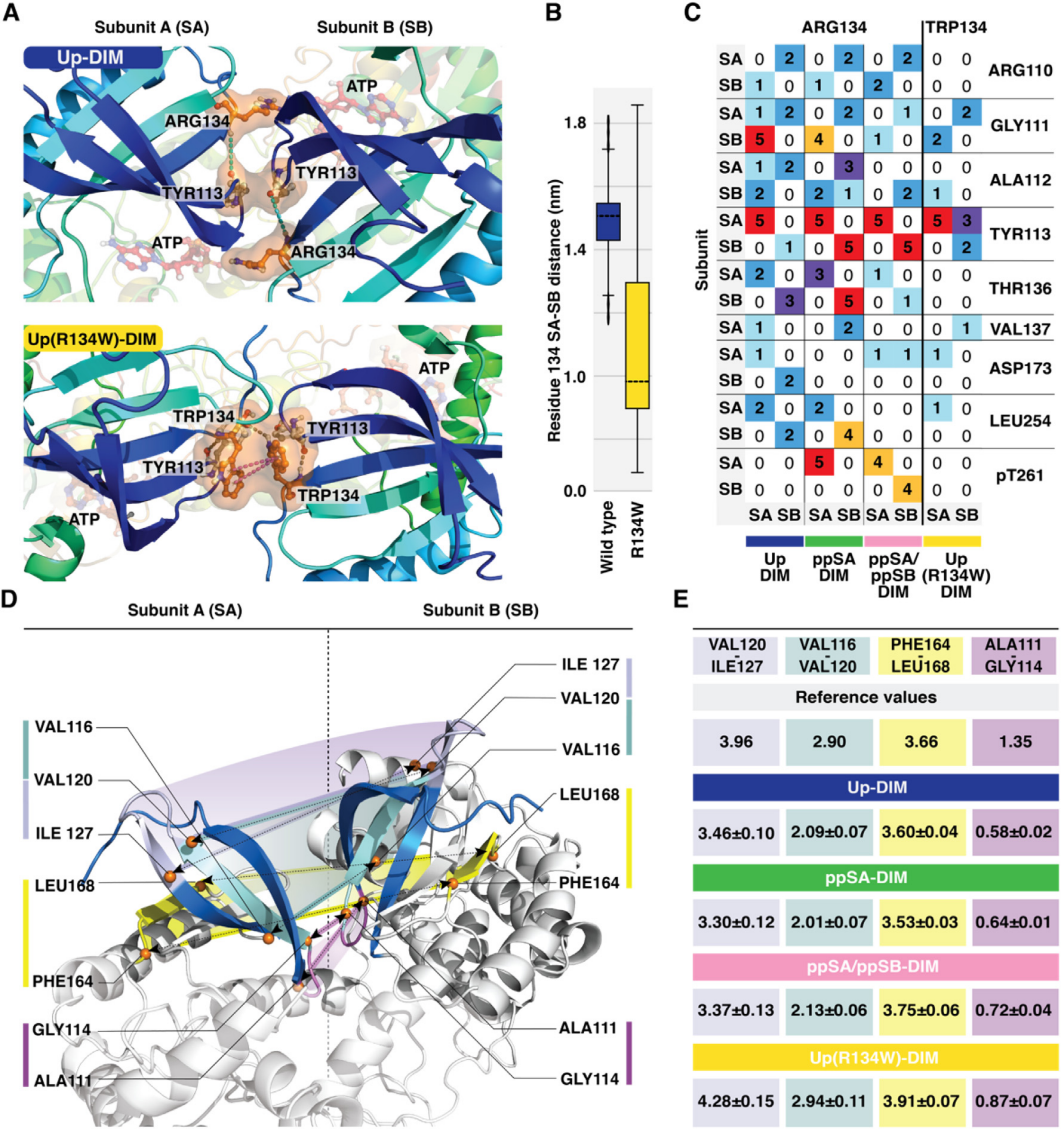
Impact of phosphorylation and R134W mutation on N-lobe interactions. (A) A representative snapshot of the top view of N-lobe, displaying difference in contact pattern between Up-DIM and Up(R134W)-DIM at 500 ns. Water-bridged interactions are indicated with dashed cyan line; hydrogen bonds with dashed grey line; π-π interaction with dashed violet line. (B) Boxplot representation of distance between the residue 134 Cα-carbons of different subunits. Simulation time of 250–1000 ns from all replicas was included in the distance analysis. The black dashed horizontal line in the box represents the median values: 1.5 nm in wild-type and 1.08 nm in R134W. Box displays the quartiles of the dataset (25–75%) and whiskers the rest of the data within 1.5 times IQR. Outliers are indicated with black diamonds. (C) Heatmap of contacts with the residue R134/W134. Numbers (0–5) represent the number of individual replicas where the interaction occurs >30% of the simulation time. (D) Selected intervals for distance analysis within dimer N-lobe interface. (E) Average distances between selected N-lobe intervals (nm) with standard deviation (SD). Reference values are calculated for 3alo crystal structure. See more details in SI Table S3. (For interpretation of the references to colour in this figure legend, the reader is referred to the web version of this article.)

Next, we evaluated the relative movement of function-related structural elements in the dimer using angle calculations as described for monomer systems (Fig. 6C). Strikingly, here the difference between the angles of unphosphorylated and phosphorylated dimer is evident. Median value of Up-DIM is close to the reference value of the crystal ~14° and for ppSA/ppSB-DIM this is clearly higher ~26°. Thus, the relative position of these structural elements is well maintained only in the unphosphorylated dimer, suggesting that the phosphorylation leads to unstable dimer. Interestingly, both the highest value and variation of the angle occur with Up(R134W)-DIM system, which clearly indicate that the mutation has influence on the unphosphorylated dimer stability and behaviour. Also, only the phosphorylation appears to distort the alpha-helical secondary structure of ASH, where in the R134W mutant this is stable as in the Up-DIM (SI Fig. S10). Similar root-mean-square deviation (RMSD) values are also observed for these structural elements in both subunits of the dimer, indicating synchronized movements between the subunits (SI Fig. S11).

The distance analysis did not reveal distortion in the dimer with the phosphorylated wild-type systems, whereas the angle calculations suggested that it clearly exists. Average end conformations of the systems reveal that in the systems where the ASH is unstable (based on angles), the dimer subunits begin to twist related to each other (SI Movies M6–8; SI Fig. S12). This explains the observation that even if the distances are comparable among the systems, the angle values reveal the distortion of the dimer. Therefore, simultaneous usage of both angle and distance calculations can provide more reliable information about protein movement. Each of these methods describe the motion along different geometric plane within the protein interface: distance calculations describe translational movements, whereas angle calculations consider rotations.

#### 3.2.3 R134W mutant distort the dimer interactions in the N-lobe

Based on the distance and angle analysis, R134W clearly affects to dynamics of the unphosphorylated dimer. This occurs especially on the N-lobe interface where R134W is located in the loop between β3-sheet and αC-helix (Fig. 5A; SI Movie M8). Therefore, we decided to study in more detail this section of MKK4 and its interactions. In the dimer, the most frequent interaction of ARG134 in wild-type MKK4 occurs with TYR113 of the same subunit, via a water bridge (Fig. 7A, 7C). This water bridge occurs in all replicas of wild-type systems with ~56% on average. Interestingly, corresponding interaction between TRP134 and backbone amino group of TYR113 of the same subunit is direct, unlike in wild-type systems where the interaction is water-mediated (Fig. 7A). Overall, we see a clear difference in a number of interactions with the R134W mutant. In four out of five replicas, the mutated residue connects the subunits TRP134(SA)–TRP134(SB) via π-π interaction (~50% frequency) and/or hydrogen bonding (17%) (Fig. 7A). In the wild-type systems, ARG134s do not show interactions between each other and are located far apart (Fig. 7B). Moreover, interaction between TRP134 from subunit B and TYR113 from subunit A occurs in three mutant replicas (Fig. 7C), providing an additional link between the subunits. In double phosphorylated monomer MKK4, ARG134 forms a salt-bridge mainly with pTHR261 (Fig. 4A), and this interaction is also frequently observed with the phosphorylated dimers.

To get more comprehensive picture of the relative movements in N-lobe, we conducted additional distance calculations with four chosen intervals (Fig. 7D). The mutant system exhibits a notable difference especially in the upper part of N-lobe (VAL120–ILE127) compared to other systems (Fig. 7E; SI Table S3). Overall, the R134W mutation disrupts local contacts in the dimer interface and clearly modifies the N-lobe association between the unphosphorylated subunits.

## 4 Discussion

Here we investigated conformational dynamics of autoinhibited MKK4 with different phosphorylation states, putative dimer stability and the effect of R134W mutation by MD simulations. The two other publicly available crystal structures of MKK4 have a disordered activation segment, where only the 3alo exists in ordered autoinhibited conformation, forming a long α-helix with unphosphorylated SER257 and THR261 (SI Fig. S1). Based on our simulations, this autoinhibited conformation of unphosphorylated MKK4 appears unstable as a monomer.

Biggest movements in the MKK4 monomer occur in the activation segment helix and in the C-lobe loop between the αF- and αH-helices. This high flexibility observed in simulations perhaps reflect to the fact why high-quality MKK4 crystal structures are unavailable. Moreover, the results here are in agreement with the recent solution structure analysis of MKK4 by SAXS [37]. Interestingly, the movement pattern of these most dynamic regions of MKK4 appears phosphorylation dependent as different phosphorylation states exhibit unique effect on MKK4 dynamics. For instance, monophosphorylated pS257 displays high fluctuation of ASH, while double phosphorylated pS257 + pT261 appears to fix ASH in a relatively stable configuration. These dynamic differences of individual phosphorylation states may reflect to the observed MKK4 activity levels (*e.g.* double phosphorylation is required for full activation) [22]. To note, there is currently no data available of the relevance and biological activity of the monophosphorylated pT261 MKK4; therefore, it may represent an artificial system.

Although the autoinhibited MKK4 conformation is unstable as monomer even when unphosphorylated, our simulation results support the possibility that this unique inactive autoinhibited state is stable as a dimer. This observation is in agreement with the results obtained from the PDBePISA assembly evaluation [47]. Our simulation results indicate that this dimer configuration is stable only when these ASH residues are unphosphorylated. In these microsecond timescale simulations, the distortion of the dimer configuration with phosphorylated or mutated MKK4 is clearly demonstrated by the angle calculations. Here a distance analysis was unable to capture the distortion. This is probably due to the fact that a full dissociation of the dimer with a clear translational movement is not expected to occur within this timescale. Interestingly, a somewhat similar type of dimerization is observed with MEK1 and MEK2 in complex with inhibitors, where allosteric inhibitors occupy partially the corresponding autoinhibited ASH position in MKK4 (SI Fig. S13) [55].

The origin of the autoinhibited MKK4 configuration is from the co-crystallized complex with ANP and p38α peptide [37]. This fact leads us to speculate the potential existence of a N-lobe binding scaffold protein which stabilizes the inactive MKK4 dimer. This scaffold protein would provide an additional regulation mechanism for MKK4́s activity. A putative candidate for this would be Scaffold protein C-Jun N-terminal kinase-interacting protein 4 (JIP4), which is known to interact with MKK4 and an increased association between these two proteins suppresses MKK4 phosphorylation [56]. This hypothesis of inactive MKK4 dimerization and the existence of a scaffold protein should be confirmed in further studies.

The most frequent MKK4 mutation in cancer, R134W, which role has not been disclosed to date, affects MKK4 dynamics on the putative inactive dimer interface. This mutation leads to dramatic alterations in the N-lobe interactions, demonstrated by the changes in frequency and nature of the interactions within the residue 134 in the N-lobe. Based on this, R134W may be an activating mutation via distorting the autoinhibited dimer state of MKK4. On the other hand, the timescale of the simulations is not sufficient to disclose a full disruption of the dimer complex. Therefore, this alteration with its shifted N-lobe interactions may even lead to enhanced stability of the inactive dimer regardless of the putative scaffold protein. This would mean that R134W is an inactivating MKK4 mutation. Overall, additional experimental evidence is required to disclose the role of this cancer associated mutation.

Our results demonstrate that the autoinhibited state of MKK4 is unstable as monomer and stable as dimer. Moreover, different phosphorylation patterns and the R134W mutation have all individual consequences for MKK4 dynamics. Better understanding of conformational changes and dimerization of protein kinases, occurring either due to phosphorylation (activation) processes or oncogenic mutations, is needed to provide comprehensive framework for disease causality. This will ensure and support a rational inhibitor design in a disease specific context related to aberrantly behaving protein kinases, which are currently the main target class in ongoing projects of the pharmaceutical industry [57].

## Author contributions

T.P. performed simulations and designed the research. E.S, A.P. and T.P. analysed the data. Original draft was written by E.S. and T.P. All authors participated in writing the final manuscript. Figures were prepared by E.S and T.P.

## Declaration of Competing Interest

The authors declare that they have no known competing financial interests or personal relationships that could have appeared to influence the work reported in this paper.

## Appendix A Supplementary data

Supplementary video M1

Supplementary video M2

Supplementary video M3

Supplementary video M4

Supplementary video M5

Supplementary video M6

Supplementary video M7
