# Supplementary material for: The autoinhibited state of MKK4: Phosphorylation, putative dimerization and R134W mutant studied by molecular dynamics simulations
Source: Comput Struct Biotechnol J. 2020 Sep 20;18:2687–98. doi: 10.1016/j.csbj.2020.09.017 (PMC7550801; doi:10.1016/j.csbj.2020.09.017)
Supplement: Supplementary data 1 [file mmc1.pdf]

SUPPLEMENTARY INFORMATION

The Autoinhibited State of MKK4: Phosphorylation, Putative Dimerization and R134W Mutant Studied by Molecular Dynamics Simulations.

Ekaterina Shevchenko[1], Antti Poso[1,2], Tatu Pantsar*[,2,3]

[1] Dept. of Internal Medicine VIII, University Hospital Tübingen, Otfried-Müller-Strasse 14, 72076 Tübingen, Germany

[2] School of Pharmacy, University of Eastern Finland, Yliopistonranta 1C, 70210 Kuopio, Finland

[3] Department of Pharmaceutical and Medicinal Chemistry, Institute of Pharmaceutical Sciences, Eberhard Karls Universität Tübingen, Auf der Morgenstelle 8, 72076 Tübingen, Germany

Content

Supplementary Figure S1........ **3**
Superimposed MKK4 structures.

Supplementary Figure S2........ **3**
RMSD values of monomer MKK4 systems.

Supplementary Figure S3........ **4**
RMSD values of homodimer MKK4 systems.

Supplementary Figure S4........ **4**
Observed angle values (αC-helix–HR–ASH) in monomer MKK4 systems.

Supplementary Figure S5........ **2**
Plane projection of residue intervals used for angle calculation and validation.

Supplementary Figure S6........ **5**
B-factor of 3alo.

Supplementary Table S1........ **5**
Notable RMSF differences (> 0.1 nm) compared to the Up system.

Supplementary Figure S7........ **6**
Principal components analysis: PC3 and PC4

Supplementary Figure S8........ **7**
Average monomer conformations at 1 μs of the simulations.

Supplementary Figure S9........ **7**
Secondary structure analysis of activation segment helix (ASH; I250–A264) in monomer MKK4.

Supplementary Figure S10........ **8**
Secondary structure analysis of activation segment helix (ASH; I250–A264) in homodimer MKK4.

Supplementary Figure S11……………………………….. **8**
RMSD for of residue intervals used for angle calculation in homodimer MKK4.

Supplementary Figure S12.………………………………………….................................. **9**
Average homodimer conformations at 1 μs of the simulations.

Supplementary Figure S13.……………………………...…….................................... **10**
Superimposed homodimer structures of MKK4, MEK1 and MEK2

Supplementary Table S2……………………….. **11**
Distances between subunit A and subunit B of MKK4.

Supplementary Table S3…………………………………………….................................. **12**
Distances within the N-lobe of dimer MKK4.

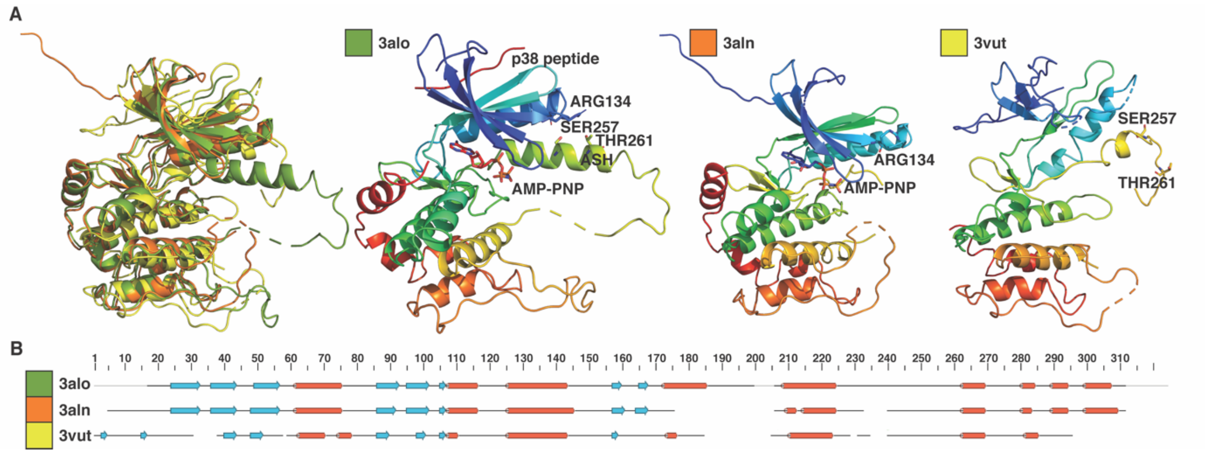

Figure S1. Superimposed MKK4 structures. (A) Three MKK4 structures are available in the RCSB Protein Data Bank (PDB IDs: 3alo, 3aln, 3vut). In the superimposed (left) structures 3alo is shown as green, 3aln as orange and 3vut as yellow. Sequence alignment **(B)** with secondary structure assignment: black lines indicate loop regions; blue arrows, β-strands; red barrels, α-helices. All structures are in inactive unphosphorylated form with length of 327 amino acids. 3alo (resolution 2.6Å) is crystallized with p38 peptide, bounded on top of N-terminal lobe of kinase. ASH (ILE250-ARG264) is in ordered autoinhibited conformation that is forming an α-helix. 3aln (resolution 2.3Å) has disordered regions: LEU254-GLY283 (alignment index 176-205), LYS309-GLY323 (alignment index 231-245). 3vut (resolution 3.5Å) is an apo structure has disordered regions: ASP263-ASP285 (alignment index 185-207), GLN316-VAL320 (alignment index 238-242), C-terminal part after ALA374 (alignment index 296) is missing.

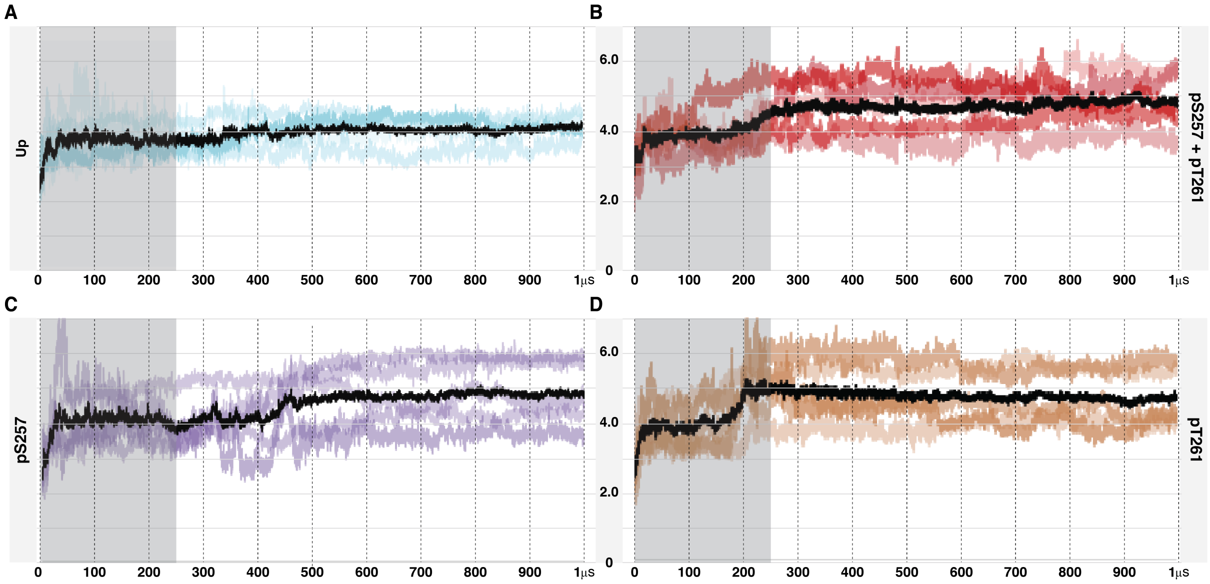

Figure S2. RMSD values of monomer MKK4 systems. (**A**) Up; (**B**) pS257+pT261; (**C**) pS257 and (**D**) pT261. RMSD was calculated for Cα-atoms. First 250 ns is highlighted with grey colour. The black line indicates the average and the other colours indicate individual replicas.

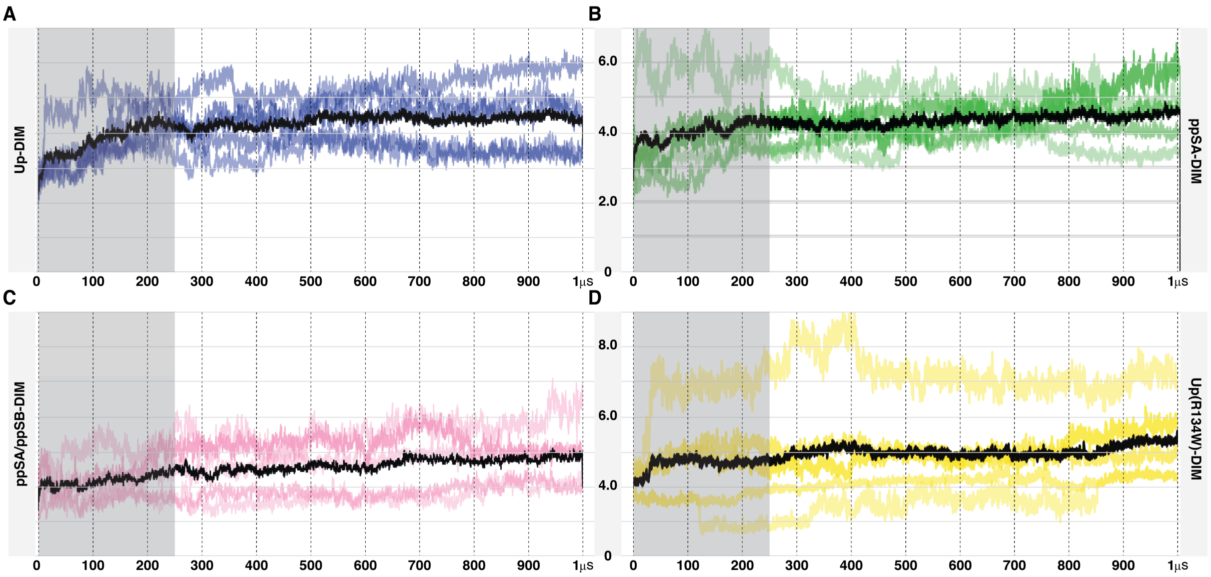

Figure S3. RMSD values of homodimer MKK4 systems. (**A**) Up-DIM; (**B**) ppSA-DIM; (**C**) ppSA/ppSB-DIM and (**D**) Up(R134W)-DIM. RMSD was calculated for Ca-atoms. First 250 ns is highlighted with grey colour. The black line indicates the average and the other colours indicate individual replicas.

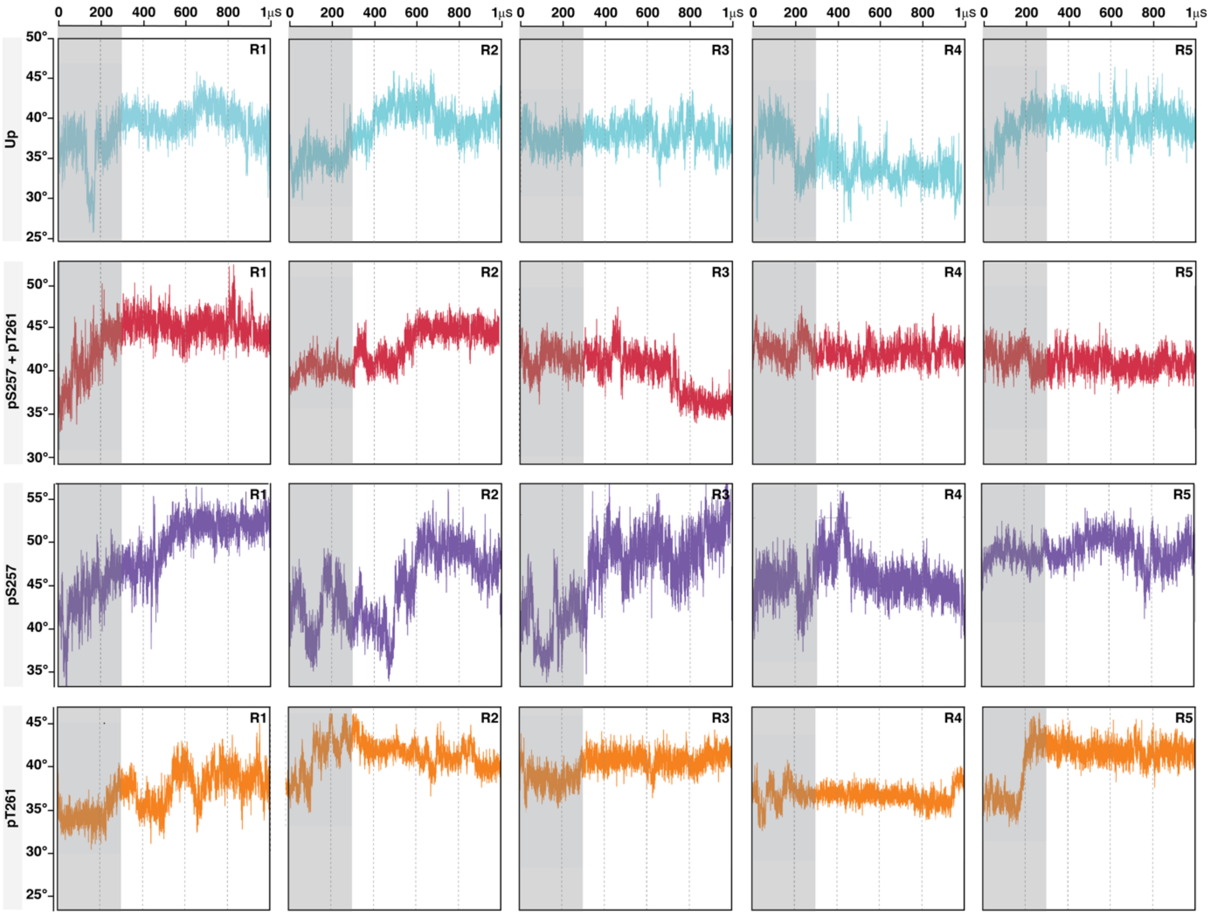

Figure S4. Observed angle values (aC-helix–HR–ASH) in monomer MKK4 systems. First 250 ns, which was excluded from the analysis related to Figure 3, is highlighted with grey colour. R1 = replica 1, R2 = replica 2, etc.

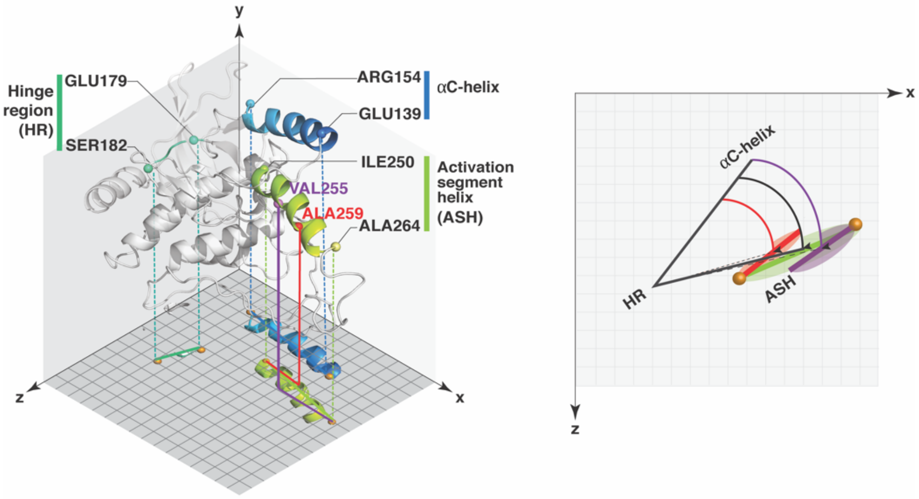

Figure S5. Plane projection of residue intervals used for angle calculation and validation. Monomer MKK4 system in three-dimensional space with flat representation of XZ-plane with different residue interval selections. Default selection of ASH defining residues ILE250–ALA264 (green) is switched to ILE250–ALA259 (red) and VAL255–ALA264 (purple). Black arrow heads on the XZ-plane indicate centre of geometry in each residue interval. Black curve indicates the reference angle (39.05°), from which red and purple display deviation of ~ -1–2° (ILE250–ALA259) and ~ +1–2° (VAL255–ALA264). Colours of selected residue intervals are consistent for both left and right images.

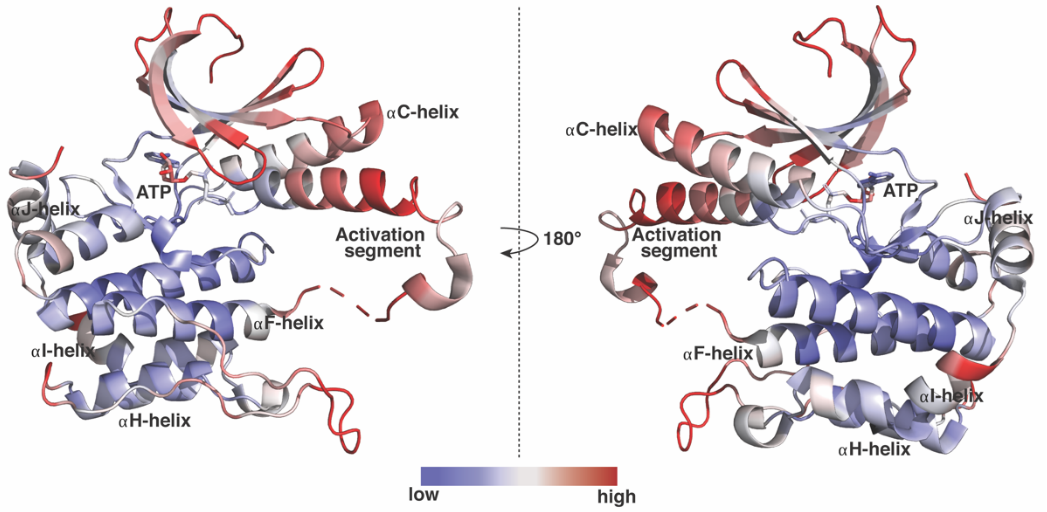

Figure S6. B-factor of 3alo crystal structure. Highest B factor values are indicated with red and lowest with blue colour. Disordered region is shown in dashed red line in the cartoon representation.

Table S1. Notable RMSF differences (> 0.1 nm) compared to the Up system. The most proximal residues of the C- and N-terminus (< ASP96 and > MET388) are excluded here.

	pS257 + pT261	pS257	pT261
**Activation loop**	K260, R262, R267–A271 (higher); I275–P277, R281, S288 (lower)	S251–K260, R262, P268–I275, S278–A279, Y284 (higher)	P268–M270, Q282–Y284 (higher)
**C-terminal loop**	P308–L317, (higher); V321, P325–L328 (lower)	W310–T318 (higher)	V313–G323 (higher)

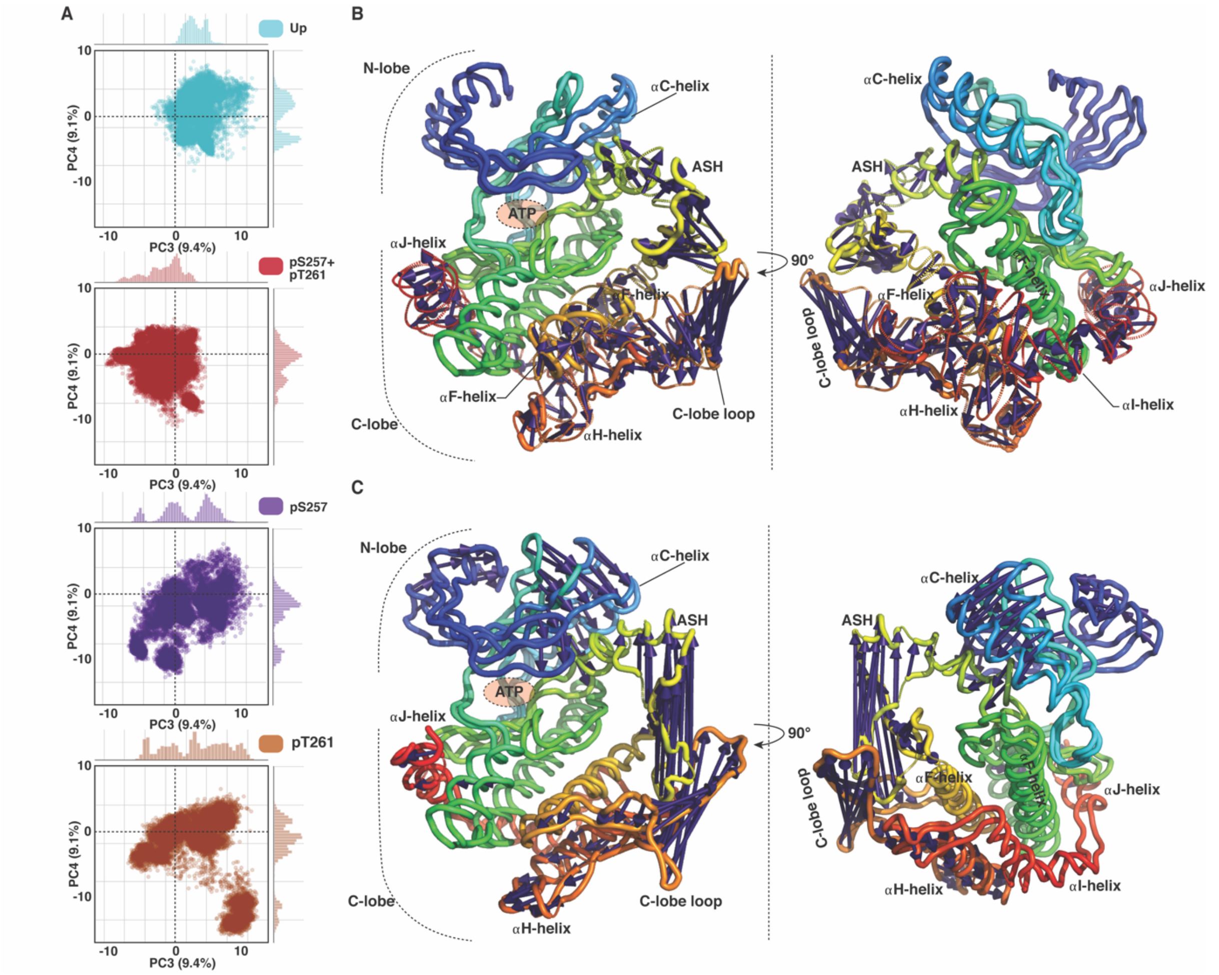

Figure S7. Principal components analysis: PC3 and PC4. (**A**) Principal component analysis (PCA) score plot of PC3 and PC4. (**B**) The extreme movements of PC3. (**C**) The extreme movements of PC4. In **B** and **C** protein is illustrated with rainbow colour and the purple arrows indicate the extreme movements.

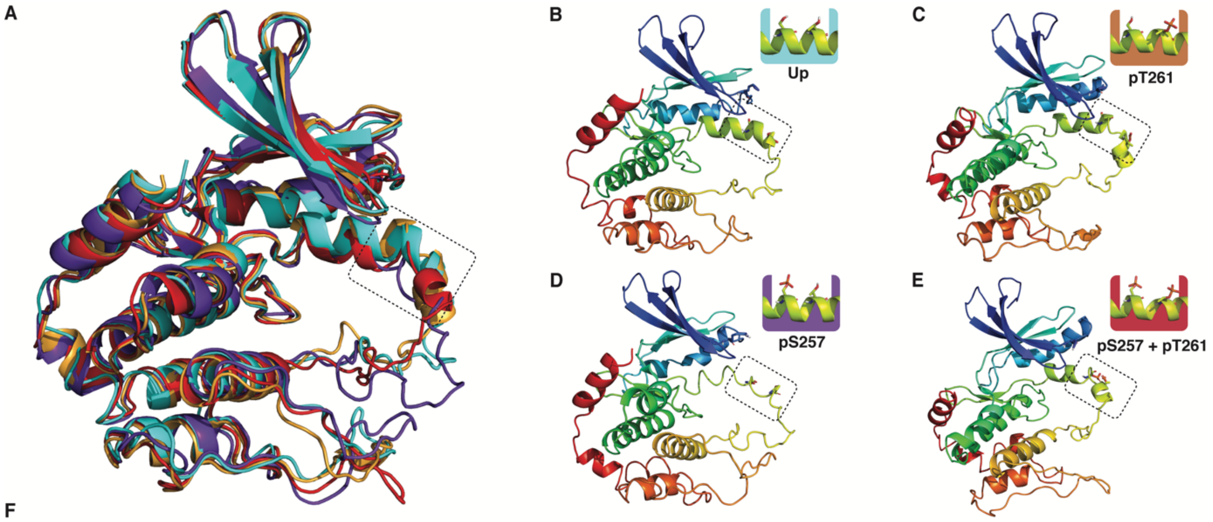

F

	Replica 1	Replica 2	Replica 3	Replica 4	Replica 5
Up	2.8	3.6	2.9	2.9	2.6
pS257	2.2	2.6	2.2	4.0	3.1
pT261	2.9	2.7	3.4	3.1	4.6
pS257+pT261	4.5	3.2	3.5	3.8	2.7

Figure S8. Average monomer conformations at 1 μs of the simulations. All systems are superimposed in **A** and each average conformation is shown as individually in **B**–**E**. Dashed square indicates position of SER257 and THR261. See also **SI Movie M5** for the conformations. (**F**) RMSD of different systems compared to their average 1 μs conformations.

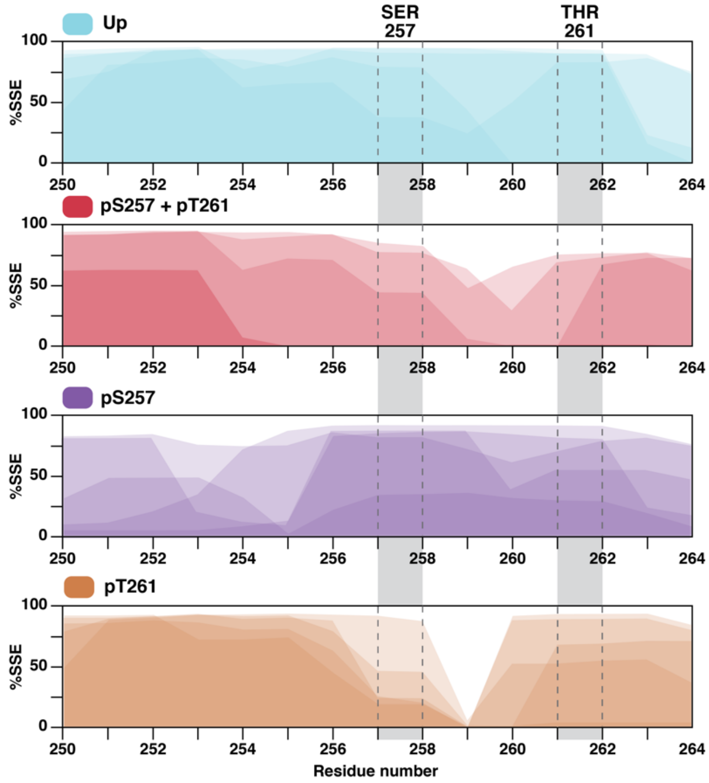

Figure S9. Secondary structure analysis of activation segment helix (ASH; I250–A264) in monomer MKK4. Plots display the percentage of time each residue contributes to each of the two secondary structure elements (%SSE): helix (coloured space inside the graphs), and loop (white space). Each replica (five replicas for each system in total) is represented with 20% colour opacity. The colour intensity is directly depended on the number of replicas, where particular secondary structure occurs. **Up**: helix 78%, loop 22%; **pS257+pT261**: helix 62%, loop 38%; **pS257**: helix 63%, loop 37%; **pT261**: helix 69%, loop 31%.

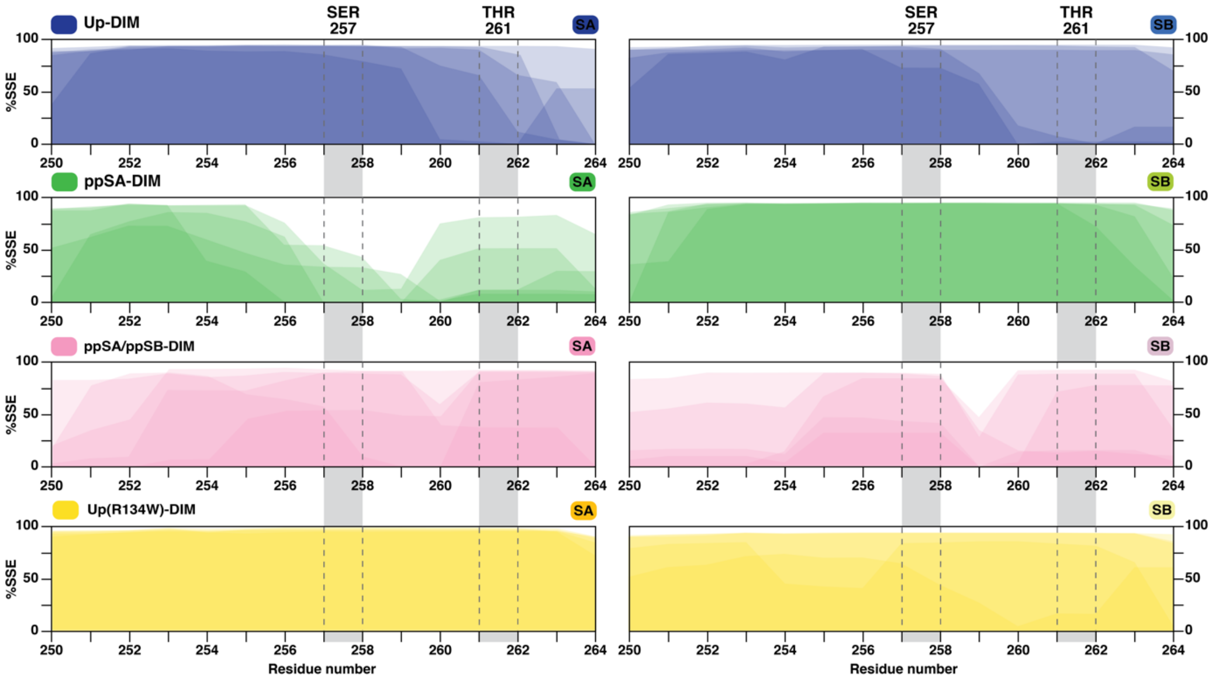

Figure S10. Secondary structure analysis of activation segment helix (ASH; I250–A264) in homodimer MKK4. Plots display the percentage of time each residue contributes to each of the two secondary structure elements (%SSE): helix (coloured space inside the graphs), and loop (white space). Analysis was performed separately for subunit A (SA) and subunit B (SB). Each replica (five replicas for each system in total) is represented on the graph with 20% colour opacity. The colour intensity is directly depended on the number of replicas, where particular secondary structure occurs. **Up-DIM** SA: helix 84%, loop 16%; SB: helix 83%, loop 17%; **ppSA-DIM** SA (phosphorylated): helix 50%, loop 50%; SB (unphosphorylated): helix 92%, loop 8%; **ppSA/ppSB-DIM** SA: helix 70%, loop 30%; SB: helix 52%, loop 48%. **Up(R134W)-DIM** SA: Helix 97%, loop 3%; SB: helix 86%, loop 14%.

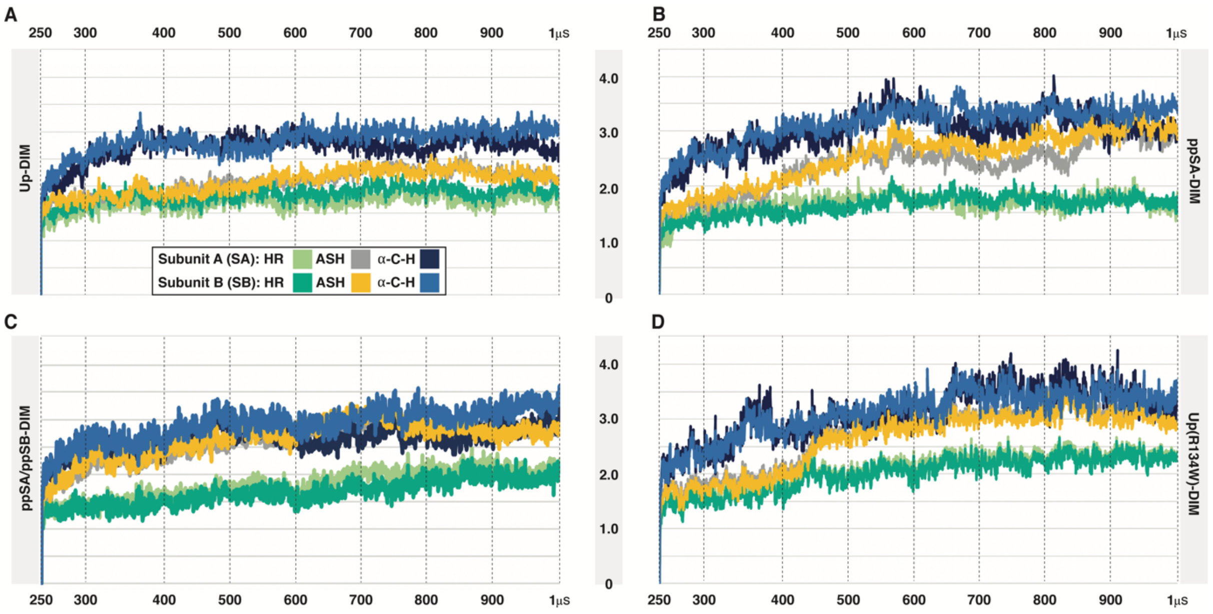

Figure S11. RMSD of residue intervals used for angle calculation in homodimer MKK4. RMSD is calculated for hinge region (HR; E179-S182; green), Activation segment helix (ASH; I250–A264; grey/yellow) and α-C-helix (α-C-H; E139–R154; blue) separately. (**A**) Up-DIM, (**B**) ppSA-DIM, (**C**) ppSA/ppSB-DIM and (**D**) Up(R134W)-DIM.

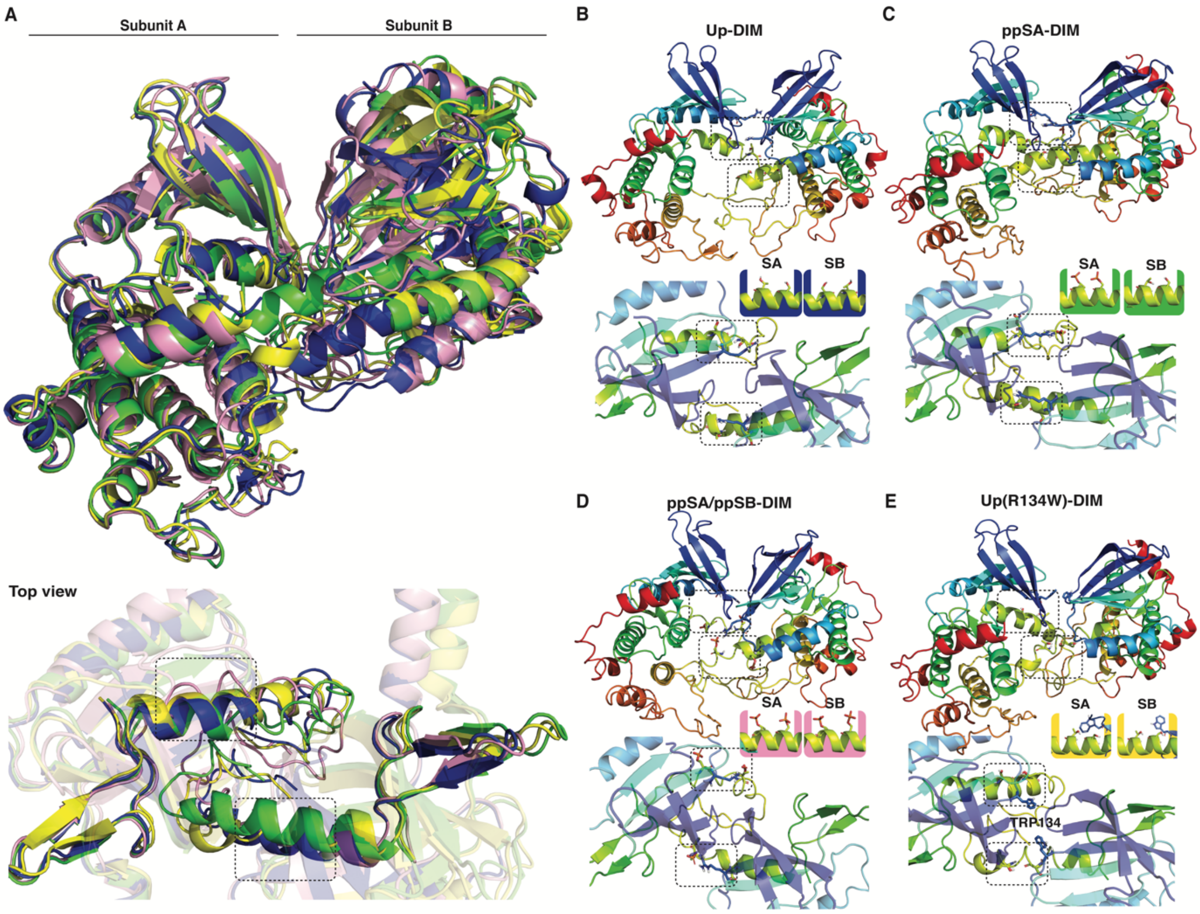

F

	Replica 1		Replica 2		Replica 3		Replica 4		Replica 5	
	SA	SB	SA	SB	SA	SB	SA	SB	SA	SB
**Up-DIM**	2.9	2.7	2.4	2.5	2.7	2.5	2.4	2.8	2.4	2.5
**ppSA-DIM**	2.5	2.4	2.9	2.5	2.6	3.5	2.4	2.6	2.2	2.5
**ppSA/ppSB-DIM**	2.5	3.0	3.0	3.0	3.0	2.7	3.4	2.3	3.3	2.7
**Up(R134W)-DIM**	2.2	2.9	2.1	2.8	2.3	2.2	2.6	3.1	2.5	2.3

Fig S12. Average homodimer conformations at 1 μs of the simulations. All systems are superimposed in **A** and each average conformation is shown as individually in **B**–**E**. Dashed square indicates position of SER257 and THR261. See also **SI Movies M6–M7** for the conformations. (**F**) RMSD of different systems compared to their average 1 μs conformations.

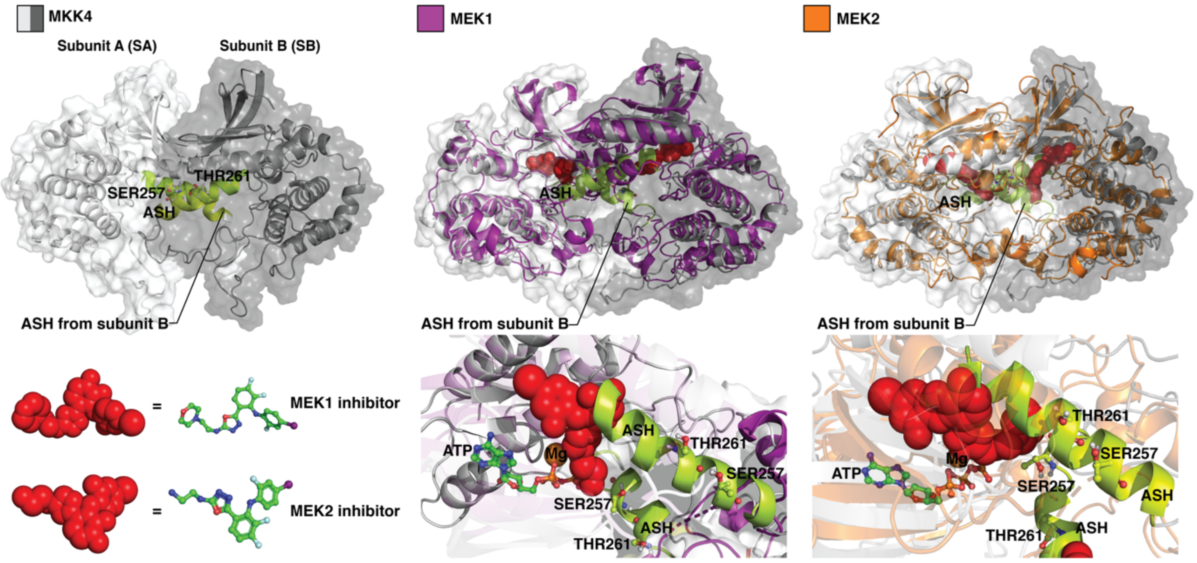

Fig S13. Superimposed structures of MKK4, MEK1 and MEK2. In the superimposed structures MKK4 is shown as grey (Subunit A) and dark grey (Subunit B), MEK1 is shown as purple, and MEK2 as orange. Activation segment helix (ASH) is shown in green. Allosteric MEK1/MEK2 inhibitors that partially occupy the corresponding ASH location in MKK4 are represented with red spheres. PDB IDs: 3alo (MKK4); 3eqb (MEK1),1s9i (MEK2).

Table S2. Distances between subunit A and subunit B of MKK4. R1 = replica 1; R2 = replica 2, etc.; SD = standard deviation

Distance (nm) Up-DIM						
Element Replica	N-lobe	SD	C-lobe	SD	Activation Segment	SD
R1	1.979	0.050	4.282	0.127	1.646	0.039
R2	2.040	0.068	3.998	0.083	1.218	0.041
R3	1.888	0.063	4.746	0.084	1.494	0.087
R4	1.901	0.047	4.035	0.078	1.496	0.043
R5	2.081	0.109	4.585	0.073	1.482	0.056
**Average**	**1.977**		**4.329**		**1.467**	
**Distance (nm) ppSA-DIM**						
Element Replica	N-lobe	SD	C-lobe	SD	Activation Segment	SD
R1	1.909	0.128	4.417	0.092	1.852	0.061
R2	1.900	0.082	4.523	0.089	1.720	0.073
R3	1.888	0.063	4.746	0.084	1.494	0.087
R4	1.890	0.058	4.631	0.080	1.423	0.036
R5	1.798	0.048	4.522	0.079	1.384	0.034
**Average**	**1.870**		**4.567**		**1.574**	
**Distance (nm) ppSA/ppSB-DIM**						
Element Replica	N-lobe	SD	C-lobe	SD	Activation Segment	SD
R1	1.877	0.061	4.205	0.087	1.514	0.066
R2	1.896	0.067	4.245	0.079	2.217	0.089
R3	1.954	0.065	4.233	0.072	1.759	0.089
R4	1.917	0.046	4.006	0.065	1.752	0.036
R5	2.382	0.125	4.322	0.057	1.706	0.047
**Average**	**2.005**		**4.202**		**1.789**	
**Distance (nm) Up(R134W)-DIM**						
Element Replica	N-lobe	SD	C-lobe	SD	Activation Segment	SD
R1	4.137	0.240	4.238	0.071	1.351	0.062
R2	2.850	0.187	4.145	0.099	1.170	0.106
R3	2.011	0.081	4.417	0.092	1.581	0.106
R4	2.635	0.068	4.376	0.135	1.510	0.036
R5	2.889	0.091	4.146	0.065	1.345	0.071
**Average**	**2.904**		**4.264**		**1.391**	

Table S3. Distances within the N-lobe of dimer MKK4. Overall table of distance statistics (nm) for each dimer system. R1 = replica 1, R2 = replica 2, etc.; SD = standard deviation

	R1		R2		R3		R4		R5		
**Distance (nm) Up-DIM**											
Distance	Average	SD	Average	SD	Average	SD	Average	SD	Average	SD	**Total average**
V116-V120	2.102	0.055	2.177	0.075	1.980	0.050	2.004	0.053	2.227	0.132	**2.098**
V120-I127	3.525	0.086	3.607	0.124	3.188	0.097	3.444	0.084	3.540	0.148	**3.461**
F164-L168	3.653	0.038	3.582	0.048	3.620	0.035	3.477	0.035	3.704	0.074	**3.607**
A111-G11	0.567	0.027	0.614	0.026	0.617	0.031	0.550	0.019	0.586	0.030	**0.587**
**Distance (nm) ppSA-DIM**											
Distance	Average	SD	Average	SD	Average	SD	Average	SD	Average	SD	**Total average**
V120-I127	3.070	0.221	3.462	0.110	3.430	0.126	3.367	0.094	3.194	0.134	**4.288**
F164-L168	3.592	0.078	3.497	0.048	3.615	0.060	3.465	0.038	3.494	0,071	**3.951**
A111-G11	0.809	0.059	0.579	0.024	0.679	0.030	0.585	0.021	0.582	0.069	**0.879**
**Distance (nm) ppSA/ppSB-DIM**											
Distance	Average	SD	Average	SD	Average	SD	Average	SD	Average	SD	**Total average**
V120-I127	3.254	0.114	3.349	0.141	3.258	0.124	3.231	0.088	3.789	0.201	**3.376**
F164-L168	3.591	0.039	3.827	0.046	3.755	0.054	3.713	0.040	3.898	0.128	**3.757**
A111-G11	0.681	0.052	0.856	0.045	0.771	0.044	0.773	0.025	0.529	0.049	**0.722**
**Distance (nm) Up(R134W)-DIM**											
Distance	Average	SD	Average	SD	Average	SD	Average	SD	Average	SD	**Total average**
V116-V120	4.390	0.244	2.177	0.075	2.148	0.091	2.878	0.067	3.121	0.093	**2.943**
V120-I127	5.895	0.268	3.607	0.124	3.439	0.114	4.100	0.120	4.398	0.134	**4.288**
F164-L168	5.032	0.155	3.582	0.048	3.554	0.073	3.786	0.049	3.804	0.070	**3.951**
A111-G11	1.650	0.1626	0.616	0.026	0.560	0.034	0.769	0.099	0.802	0.069	**0.879**
